# Molecular subtypes based on cuproptosis-related genes and tumor microenvironment infiltration characteristics in pancreatic adenocarcinoma

**DOI:** 10.1186/s12935-022-02836-z

**Published:** 2023-01-16

**Authors:** Jian Li, Jingyang Yin, Wenhua Li, Huaizhi Wang, Bing Ni

**Affiliations:** 1 Department of Pathophysiology, College of High Altitude Military Medicine, Third Military Medical University, Chongqing, 400038 People’s Republic of China; 2 Department of General Surgery, Air Force Hospital of Western Theater Command, Chengdu, 610021 People’s Republic of China; 3 Institute of Hepatopancreatobiliary Surgery, Chongqing General Hospital, Chongqing, 400038 People’s Republic of China; 4 Department of Cadre Ward, Air Force Hospital of Western Theater Command, Chengdu, 610021 People’s Republic of China

**Keywords:** Cuproptosis, Pancreatic adenocarcinoma, Molecular subtypes, Tumor microenvironment, Immunotherapy

## Abstract

**Background:**

Multiple molecular subtypes with distinct clinical outcomes in pancreatic adenocarcinoma (PAAD) have been identified in recent years. Cuproptosis is a new form of cell death that likely involved in tumor progression. However, the cuproptosis-related molecular subtypes as well as its mediated tumor microenvironment (TME) cell infiltration characteristics largely remain unclear.

**Methods:**

Expression profiles of 10 cuproptosis-related genes (CRGs) and their association with patient survival, TME, cancer stemness and drug resistance were studied in 33 cancer types using the TCGA pan-cancer data. Using 437 PAAD samples from five cohorts (TCGA-PAAD cohort and four GEO cohorts), we explored the molecular subtypes mediated by CRGs, along with the associated TME cell infiltration. Unsupervised methods were utilized to perform cuproptosis subtype clustering. The cuproptosis score was constructed using the COX regression model with least absolute shrinkage and selection operator regression (LASSO) algorithm to quantify the cuproptosis characteristics of a single tumor.

**Results:**

The expression of 10 CRGs varies in different cancer types with striking inter- and intra- cancer heterogeneity. We integrated the genomic profiling of the CRGs and identified three distinct cuproptosis subtypes, and found that multi-layer CRG alterations were correlated with patient prognosis and TME cell infiltration characteristics. In addition, a cuproptosis score signature was constructed to predict prognosis, and its clinical impacts were characterized in the TCGA-PAAD cohort. The cuproptosis signature was significantly associated with prognosis, tumor subtypes, CD8 T-cell infiltration, response to immune checkpoint inhibitors (ICIs) and chemotherapeutic drug sensitivity. Furthermore, the expression patterns of CRGs in pancreatic cancer cells and normal controls were validated, which was almost consistent with the results from the public database. The expression level and prognostic predictive capability of DLAT were verified in 97 PAAD patients from our patient cohort.

**Conclusions:**

These findings may help understand the roles of CRGs in PAAD and the molecular characterization of cuproptosis subtypes. In addition, the cuproptosis score could serve as a promising biomarker for predicting prognosis and response to immunotherapy in PAAD patients.

**Supplementary Information:**

The online version contains supplementary material available at 10.1186/s12935-022-02836-z.

## Background

Pancreatic adenocarcinoma (PAAD) is among the most lethal malignancies, with a 5-year survival rate of approximately 10% [1]. Worldwide, the health burden of PAAD is increasing annually, and PAAD is projected to become the second leading cause of cancer-related death by 2030 [2]. Many factors, such as its broad resistance to therapy, lead to a dismal prognosis; however, efficient treatment remains limited [3]. Cumulative evidence has revealed that the current clinical classification of PAAD has limitations for efficient treatment and accurate prognostic prediction because of the strong heterogeneity discovered in PAAD [4, 5]. In recent years, with the development of tumor genomics, an accumulating number of molecular subtypes have been investigated to guide the treatment and predict the prognosis of PAAD. Collisson et al. [6] identified three PAAD subtypes, classical, quasi-mesenchymal and exocrine-like, and evidenced the differences in prognosis and therapeutic response between them. Bailey et al. [7] classified PAAD into four molecular subtypes correlated with histopathological characteristics and clinical outcomes based on a large cohort of 456 PAAD patients. Despite our deepened understanding of the molecular subtypes of PAAD, the prognostic prediction of patients with PAAD is less satisfactory, suggesting that there are still immense differences within the molecular subtypes. Therefore, more prognostically related factors need to be considered to stratify patients more precisely.

As an essential cofactor for all organisms, copper homeostasis plays crucial roles in various physiological processes [8]. Genetic variation in copper homeostasis can even lead to life-threatening diseases, including Wilson's diseases and neurodegenerative disorders [9, 10]. Furthermore, imbalances in copper homeostasis have been shown in many malignancies [11, 12]. Recent studies have demonstrated that many kinds of copper ionophores can act as anticancer agents, such as disulfiram [13, 14], elesclomol [15] and dithiocarbamates [16]. Interestingly, some copper ionophores have been disclosed to have an intrinsic selectivity in preferentially killing cancer cells rather than normal cells, which showed promise for innovative anticancer treatment [17]. In addition, some copper chelators have also been suggested as antitumor drugs [18–20]. Excitingly, Tsvetkov et al. recently discovered a novel type of copper-induced cell death distinct from all other known mechanisms of cell death and defined it as cuproptosis [21]. In their study, they demonstrated that copper can directly bind to lipoylated components of the tricarboxylic acid (TCA) cycle, leading to lipoylated mitochondrial protein aggregation and subsequent iron-sulfur cluster protein loss. This triggered proteotoxic stress and ultimately cell death [21]. Ten cuproptosis-related genes (CRGs) were also identified, among which seven were positive hits and three were negative hits. However, the role of cuproptosis in tumor progression is not well defined, and the comprehensive role of these CRGs in PAAD phenotyping and the tumor microenvironment (TME) is not clear.

In this study, 437 PAAD samples were divided into three discrete cuproptosis subtypes based on CRG expression levels, and the prognosis and immune infiltration differences among the subtypes were examined. Samples were then classified into three gene clusters according to differentially expressed genes (DEGs) identified in different cuproptosis subtypes. Finally, a cuproptosis score was established to predict prognosis and characterize the immune landscape of PAAD.

## Methods

### Data collection and preprocessing

We collected gene expression profiles of 177 PAAD samples from the TCGA-PAAD cohort (https://gdc.cancer.gov) and 277 PAAD samples from four cohorts (GSE62452, GSE28735, GSE21501 and GSE57495) in the Gene Expression Omnibus (GEO) repository (https://www.ncbi.nlm.nih.gov/geo/). The raw “cel” files were downloaded from the GEO dataset, and background adjustment and quantitative normalization were performed. Transcriptome data (FPKM value) of TCGA-PAAD were transformed into transcripts per kilobase million (TPM) and were supposed to be identical to those from microarrays as previously reported [22]. Next, we performed the “Combat” algorithm to remove the batch effect of the merged dataset from the five cohorts. We excluded data from patients without survival information or with a survival time less than 30 days; thus, 437 PAAD samples were included in the subsequent analyses. In addition, 167 normal pancreas samples were collected from the Genotype-Tissue Expression (GTEx) database to obtain the expression profiles of 10 CRGs between PAAD and normal samples; the TCGA-PAAD cohort had only four normal samples.

### Pan-cancer analysis of the CRGs

TCGA pan‒cancer data, including RNA-Seq, clinical data, immune subtypes and stemness scores based on mRNA (RNAss) and DNA methylation (DNAss), were downloaded from the Xena platform (https://xenabrowser.net/datapages/). Expression comparison (Mann‒Whitney test) and survival analysis (univariate Cox regression) of the CRGs were implemented based on TCGA pan‒cancer data. The expression data of the immune score and stromal score from ESTIMATE [23] were utilized to analyze TME infiltration in different tumors. In addition, the six immune subtypes were used to examine the relationship between CRGs and immune subtypes by analysis of variance. Spearman correlation between the cancer stemness indices (RNAss and DNAss) and each CRG was performed to evaluate whether CRGs influence cancer stemness.

### NCI-60 analysis

The NCI-60 human tumor cell line screen is a National Cancer Institute (NCI) 60 cancer cell line anticancer drug discovery project, which can be accessed via the CellMiner interface (https://discover.nci.nih.gov/cellminer/). To evaluate the association between CRGs and drug sensitivity, Pearson correlation was performed between each CRG expression and the z scores of cell sensitivity data (GI50) after drug treatment. The drug responses of 262 FDA-approved drugs that are currently in clinical trials were used in the correlation analysis.

### Unsupervised clustering for CRGs

Initially, ten CRGs were collected from previous studies [21], and six CRGs were selected via survival analysis. Then, unsupervised cluster analysis was utilized to classify the patients based on the expression of six CRGs. The optimal number of stable subtypes was determined by the consensus clustering algorithm. We performed these steps based on the ConsensusClusterPlus R package and repeated it for 1000 cycles to ensure the stability of the classification.

### Functional and pathway enrichment analysis

Gene set variation analysis (GSVA) was performed to investigate the biological difference between different subtypes using the “GSVA” R package. The “c2.cp.kegg.v7.2.symbols” gene sets were downloaded from the Molecular Signatures Database (MSigDB) to run GSVA. To explore the potential functions of DEGs among the three cuproptosis clusters, functional enrichment analyses were implemented on the DEGs using the clusterProfiler R package. Gene set enrichment analysis (GSEA, version 4.2.3) was performed to analyze the high and low cuproptosis score groups and explore the possible cellular pathways.

### Correlations of molecular subtypes with TME in PAAD

To understand the immune status of the PAAD samples, the CIBERSORT algorithm was used to evaluate the relative proportion of 22 immune cells. We utilized the ESTIMATE algorithm to evaluate the immune and stromal scores of each patient. The tumor immune estimation resource (TIMER), CIBERSORT, CIBERSORT-ABS, QUANTISEQ, microenvironment cell populations-counter (MCP-COUNTER), XCELL and estimating the proportion of immune and cancer cells (EPIC) algorithms were used to evaluate the abundances of immune cells between the high and low cuproptosis score groups based on the cuproptosis signature. Pearson correlation analysis was performed to assess the correlation of the cuproptosis score and immune infiltration. Furthermore, the single-sample GSEA (ssGSEA) algorithm was applied to measure the immune functions and immune cell infiltration between the two cuproptosis score groups. We also examined the correlations between the cuproptosis subtypes and the expression levels of immune checkpoint genes.

### Identification of the DEGs

The DEGs were identified using the limma R package, and genes with an adjusted P value < 0.001 were the DEGs of different cuproptosis clusters. To further explore the potential interactions of cuproptosis pattern-related DEGs, a protein–protein interaction (PPI) network was constructed using the STRING database [24].

### Construction of the cuproptosis prognostic signature

First, 40 prognostic DEGs among the cuproptosis clusters were identified by univariate Cox analysis. Then, a multivariate Cox regression model with least absolute shrinkage and selection operator (LASSO) penalties was performed to obtain the optimal prognostic signature. As a result, 10 hub genes and their correlative coefficients were obtained to develop the cuproptosis prognostic signature, defined as the cuproptosis score. The cuproptosis score of each sample was obtained by taking the sum of the expression of each gene and multiplying it by the corresponding coefficients for each sample. Using the median score as the cutoff point, PAAD patients were divided into a high score group and a low score group. Survival analysis was applied to analyze the differences in overall survival (OS) between the two groups using the R package “survminer”. Receiver operating characteristic (ROC) curves were plotted by the R package “timeROC”, and AUCs at different time points were evaluated to confirm the diagnostic value of the score signature. In addition, principal component analysis (PCA) was implemented using the “Rtsne” and “ggplot2” packages to evaluate the classification ability of the signature. Independent analysis for the cuproptosis score was performed by univariate and multivariate Cox regression. The hazard ratio (HR) and 95% confidence interval (CI) were calculated. We also constructed a prognostic signature nomogram based on the R package “rms”. The calibration curve was plotted to determine the fitting and predictive ability of our prognostic model. Finally, we conduct decision curve analysis (DCA) to evaluate the net benefits with the different predictors using the “rmda” package.

### Immunophenoscore analysis

The immunophenoscore (IPS), calculated based on the four main types of genes that determine immunogenicity, has the ability to predict the patients’ response to immune checkpoint inhibitors (ICIs) [25]. The IPS range is between 0 and 10. A higher score indicates stronger immunogenicity and a better response to ICIs. The IPSs of TCGA-PAAD patients were downloaded from The Cancer Immunome Atlas (TCIA).

### Mutation and drug susceptibility analysis

To determine the somatic mutations of PAAD patients between high and low cuproptosis score groups, the mutation annotation format (MAF) from the TCGA database was generated and then visualized using the “maftools” R package. We also evaluated the tumor mutation burden (TMB) for each PAAD sample in the two groups. To assess the therapeutic effects of chemotherapy drugs in the two groups, we evaluated the half-maximal inhibitory concentration (IC50) values of chemotherapy drugs commonly used to treat PAAD patients using the “pRRophetic” package.

### Cell lines and tissue samples

The human pancreatic cancer cell lines AsPC-1, BxPC-3, CFPAC-1 and PANC-1 (ATCC, Manassas, USA) and the human pancreatic ductal epithelial cell line HPDE6-C7 (BeNa Culture Collection, China) were cultured in complete growth medium, as recommended by the manufacturer. Ninety-seven paraffin-embedded, archived PAAD samples (10 with adjacent nontumorous tissues) used in the study were histopathologically and clinically diagnosed at the Institute of Hepatopancreatobiliary Surgery, Southwest Hospital, Third Military Medical University between 2012 and 2015. Normal pancreas tissues were obtained from organ donors. Written informed consents were obtained from all patients prior to the study. None of the patients had received radiotherapy or chemotherapy before surgery. The use of clinical specimens for research purposes was approved by the ethical committee of Southwest Hospital (approval no. 20220308).

### Quantitative real-time PCR (qRT‒PCR)

Total RNA was extracted from cancer cell lines using an Ultrapure RNA kit (Cwbio, Beijing, China). Reverse transcription and real-time PCR were implemented using a PrimeScript RT reagent kit and SYBR® Premix Ex Taq™ kit (Takara, Dalian, China) according to the manufacturer's instructions. The 2^−ΔΔCt^ method was used to calculate the relative mRNA expression levels. The primers used in this study are presented in Additional file 2: Table S1.

### Immunohistochemistry

Immunohistochemistry (IHC) analysis was performed as described in our previous reports [26]. Briefly, after blocking, the sections were incubated overnight with anti-DLAT antibody (1:100; Proteintech, Chicago, USA), followed by incubation with a secondary antibody and then cultivation with a streptavidin–biotin complex (Maixin, Fuzhou, China). The IHC scores were determined by combining the percentage of positively stained cancer cells (0, 0%; 1, < 10%; 2, 10–50%; 3, > 50%) and the intensity of staining (0, no staining; 1, weak staining; 2, moderate staining; 3, strong staining). For statistical analyses, the cancer samples were grouped into those with low expression (≤ 4) and high expression (≥ 6).

### Western blotting

Western blotting was performed as previously described [26] using antibodies targeting DLAT (1:1000; Proteintech, Chicago, USA) and GAPDH (1:2000; Cell Signaling Technology, Boston, USA). The horseradish peroxidase-conjugated antibody was used as the secondary antibody (1:2000; Cell Signaling Technology). Protein levels were normalized against the endogenous control.

### Statistical analysis

All computational and statistical analyses were performed using R version 4.1.0. The difference between two groups was examined using the Wilcoxon test. The Kruskal‒Wallis and one-way ANOVA tests were used to analyze the significance of differences among the three groups. The “ggalluvial” package was used to obtain the alluvial diagram. In comparisons between groups, statistical significance was set at P < 0.05. *P < 0.05; **P < 0.01; ***P < 0.001.

## Results

### Pan-cancer analysis

**Fig. 1:**
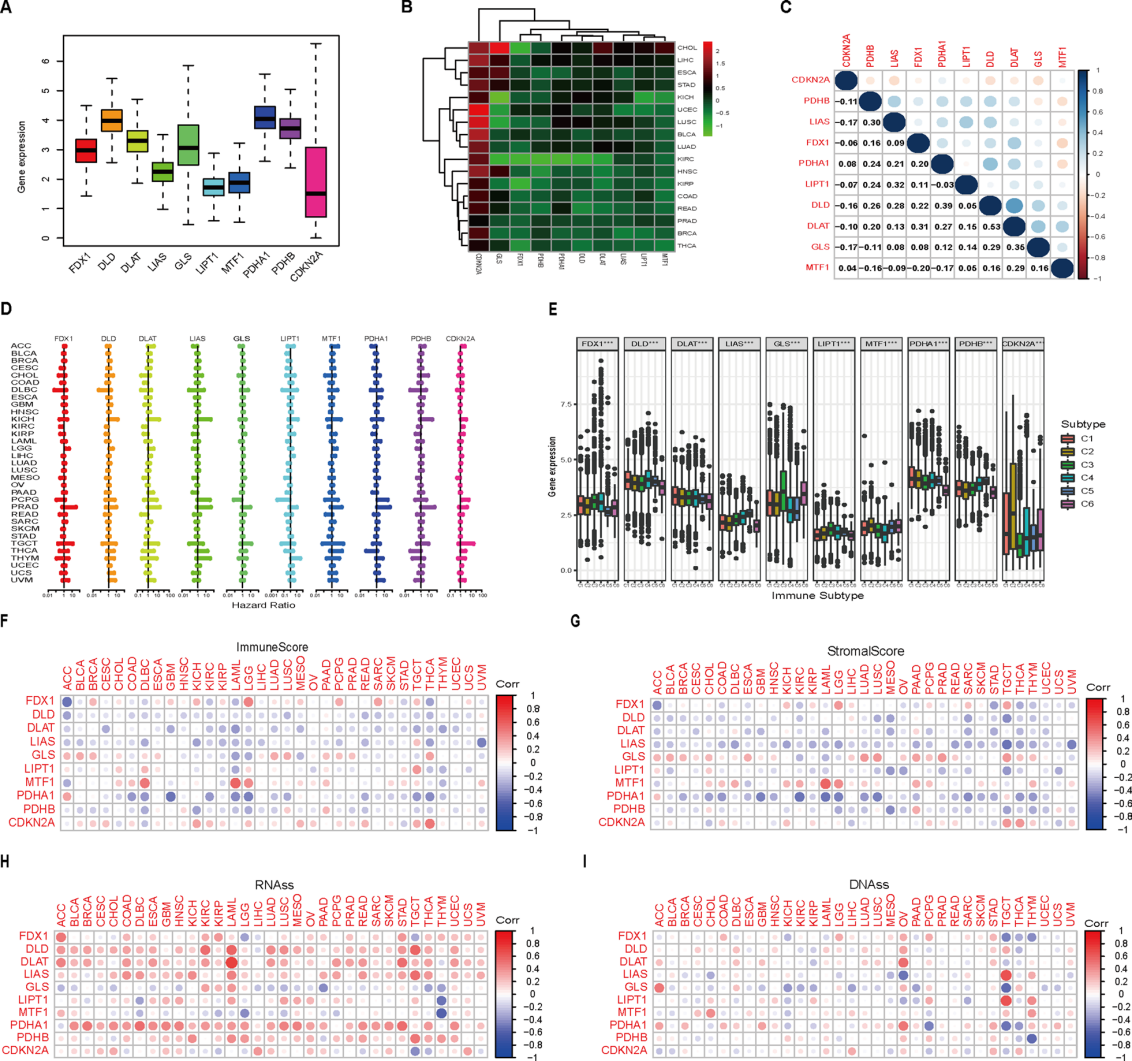
Pan-cancer analysis of the 10 CRGs. **A** The expression level of CRGs across all 33 tumor types. **B** Heatmap of CRGs between tumor and adjacent normal tissues for 17 tumor types that have more than five adjacent normal samples. **C** The correlation of gene expression among the 10 CRGs across cancers based on the Spearman correlation test. **D** Forest plots for hazard ratios of CRGs in different cancer types. **E** The association between CRGs and immune subtypes. **F**, **G** The relationship between the ESTIMATE immune score, stromal score and CRG expression. **H**, **I** The association between cancer stemness features and CRG expression based on RNAss and DNAss

We first curated a catalog of 10 CRGs that function closely with cuproptosis [21], of which seven were positively regulated (FDX1, LIAS, LIPT1, DLD, DLAT, PDHA1 and PDHB) and three were negatively regulated (MTF1, GLS and CDKN2A). To study the intrinsic expression pattern of the CRGs, we examined their expression levels in all 33 cancer types available in TCGA pan-cancer data. The expression levels of 10 CRGs showed prominent inter-tumor heterogeneity across different cancer types (Fig. 1A). Gene differential expression analysis indicated that the 10 CRGs were differentially expressed in tumor tissues compared with corresponding normal tissues (Fig. 1B and Additional file 1: Fig. S1). For instance, the expression levels of GLS showed the largest inter-tumor heterogeneity, with some tumors having very low levels of GLS (KICH, UCEC, LUSC and KIRC), while CHOL had a clearly high level of GLS expression (Fig. 1B). However, as a tumor suppressor gene [27], the expression levels of CDKN2A were upregulated in many cancer types (Fig. 1B). Spearman correlation suggested that DLAT and DLD had the highest correlation (r = 0.53, P < 0.0001), suggesting that these two genes may have some potential interaction (Fig. 1C). For survival analysis, we found that the same CRG had different prognostic significance in distinct cancer types (Fig. 1D). For example, CDKN2A was positively associated with OS in MESO but negatively associated with OS in LIHC, ACC, PCPG and UCEC (Additional file 1: Fig. S2). It is speculated that CRGs may affect the sensitivity of tumors to antineoplastic agents by inducing cuproptosis. Next, using drug screened genomic data from the NCI-60 panel, we investigated the association between CRGs and drug sensitivity. The top nine gene-drug pairs are shown in Additional file 1: Fig. S3, and the whole list of the drug sensitivity analysis results is shown in Additional file 2: Table S2. Interestingly, we found that the seven cuproptosis positive hits were mainly positively related to chemotherapy sensitivity. Conversely, the cuproptosis negative hits, including MTF1, GLS and CDKN2A, were mostly negatively related to drug sensitivity. FDX1, a key regulator of cuproptosis [21], has been shown to be positively related to eight drug sensitivities, including ifosfamide, oxaliplatin, chelerythrine, PX-316, pyrazoloacridine, 7-hydroxystaurosporine, amonafide and nelarabine. All these drugs have been reported to have antitumor activity, among which ifosfamide is a cell cycle nonspecific drug and has inhibitory effects on a variety of tumors, and oxaliplatin is a commonly used platinum-based chemotherapy drug for the treatment of colorectal cancer. These findings suggested that cuproptosis might participate in some antitumor treatments.

Six immune subtypes have been defined in human tumors, including C1 (Wound healing), C2 (INF-γ dominant), C3 (Inflammatory), C4 (Lymphocyte depleted), C5 (Immunologically quiet), and C6 (TGFβ dominant) [28]. As shown in Fig. 1E, the levels of 10 CRGs were differentially expressed across different immune subtypes in the pan-cancer data (all P < 0.001). In addition, TME analysis showed that the CRGs are involved in immune and stromal cell infiltration in most tumor types (Fig. 1F, G). These results suggested that the cuproptosis process is linked to changes in the TME and immune infiltrate. Finally, we performed tumor stemness feature analysis. Tumor stemness can be examined based on RNAss (mRNA expression) and DNAss (DNA-methylation pattern) [29]. The CRGs showed different levels of association with RNAss and DNAss across diverse cancer types (Fig. 1H, I). Interestingly, we found that there was striking heterogeneity in the correlation between CRGs and tumor stemness in different tumor types, with some tumor types expressing a very high positive correlation, while others expressed a clearly negative correlation of that gene. For example, CDKN2A positively correlates with RNAss and DNAss in LIHC, while it showed a significantly negative correlation with tumor stemness in TGCT and KIRP. In addition, almost all the CRGs were strongly positively correlated with RNAss for KICH; however, most CRGs showed a negative correlation with DNAss in KICH. These contradictory results showed that RNAss and DNAss may identify different cancerous cell populations featuring different characteristics or degrees of stemness.

### Genetic and transcriptional alterations of CRGs in PAAD

**Fig. 2:**
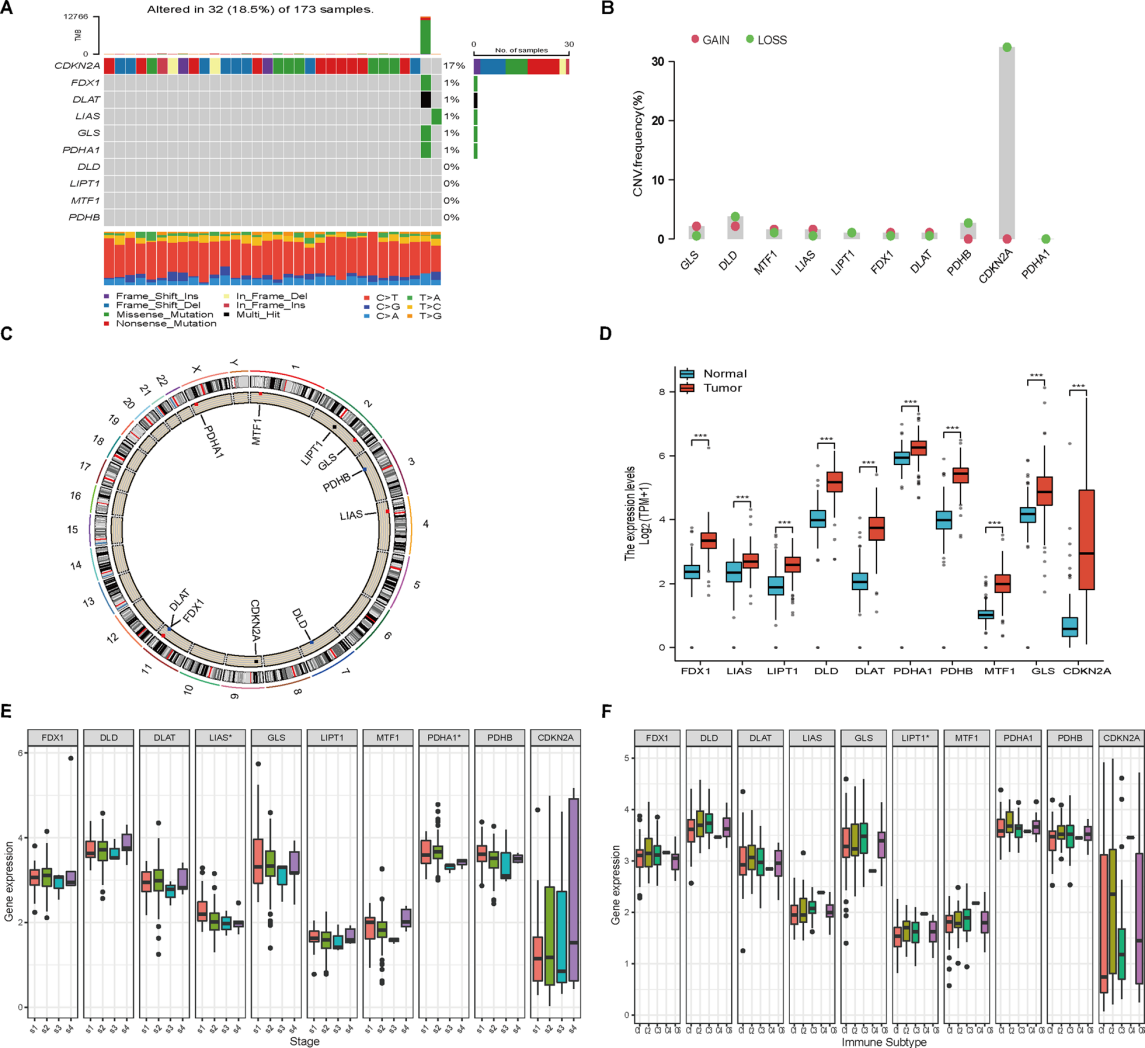
Genetic and transcriptional alterations of CRGs in PAAD. **A** Mutation frequencies of the 10 CRGs in 173 patients from the TCGA-PAAD dataset. **B** Frequencies of CNV gain and loss among CRGs. **C** The locations of the CNV alterations in the CRGs on their respective chromosomes. **D** Expression distributions of CRGs between normal and PAAD tissues in the TCGA-GTEx cohort (Wilcoxon rank sum test, ***P < 0.001). **E** The association between CRGs and different tumor stages. **F** The association between CRGs and different immune subtypes

First, the landscape of mutation profiles in 173 pancreatic cancer patients from the TCGA-PAAD cohort was analyzed. The results showed that 32 of the 173 samples (approximately 18.5%) showed CRG mutations. Of these, CDKN2A showed the highest frequency of mutations (approximately 17%). However, no mutation was identified in four CRGs (DLD, LIPT1, MTF1 and PDHB) (Fig. 2A). As CDKN2A showed the highest mutation frequency, we evaluated the relationship between CDKN2A mutation and CRG expression. The results showed that the expression levels of CDKN2A were significantly associated with CDKN2A mutation status (Additional file 1: Fig. S4A). In addition, KRAS is the most common oncogene and has been found to be mutated in approximately 90% of PAAD cases. Hence, we also analyzed the relationship between KRAS mutation and CRG expression. The results revealed that the expression levels of FDX1 and DLAT were significantly associated with KRAS mutation status (Additional file 1: Fig. S4B). Next, we examined somatic copy number alterations in these CRGs and identified some copy number alterations in all the CRGs. Among them, GLS, MTF1 and LIAS had copy number variation (CNV) increases, while CDKN2A showed a substantial CNV decrease (Fig. 2B). The location of CNV alterations of CRGs on chromosomes is shown in Fig. 2C. We further examined the relationship between CNV alterations and CRG expression levels in PAAD. Figure 2D shows that all the CRGs were significantly elevated in PAAD samples. However, the CNV increase in the CRGs was not obvious in PAAD, suggesting that CNV might not regulate the mRNA expression of CRGs. There may be other factors involved in the regulation of CRG expression that need to be detected. In addition, the association between CRG expression levels and different tumor stages and immune subtypes was analyzed in PAAD. As shown in Fig. 2E, F, LIAS and PDHA1 were expressed differently in diverse clinical stages (P < 0.05), while LIPT1 was expressed differently among immune subtypes (P < 0.05). Finally, Pearson correlation showed that the CRGs had different levels of association with tumor stemness and TME in PAAD (Additional file 1: Fig. S5).

### Identification of cuproptosis subtypes in PAAD

**Fig. 3:**
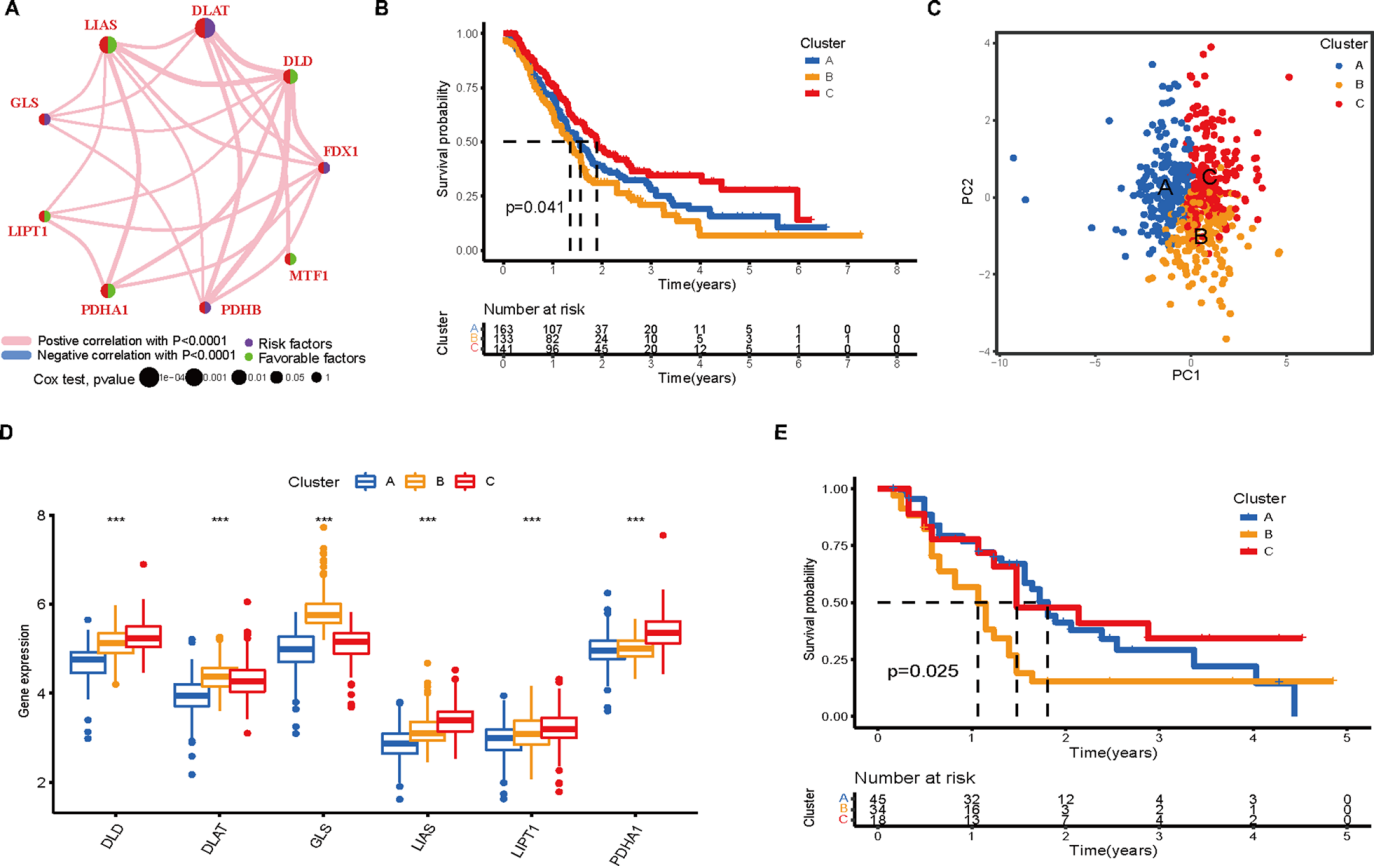
Identification of cuproptosis subtypes in PAAD. **A** Interactions among CRGs in PAAD. The size of the circle represents the impact of each CRG on the prognosis; the P value was evaluated by the log-rank test. Green dots represent protective factors, and violet dots represent risk factors. **B** Kaplan‒Meier curves of the three cuproptosis subtypes. **C** PCA identified three distinct subtypes based on the expression levels of CRGs. **D** Differences in the expression of CRGs among the three cuproptosis subtypes (***P < 0.001). **E** Survival analysis of the GSE21501 cohort with three cuproptosis subtypes

To fully understand the expression pattern of CRG involved in tumorigenesis, five eligible PAAD cohorts (TCGA-PAAD, GSE62452, GSE28735, GSE21501 and GSE57495) were integrated in our study for further analysis (Additional file 2: Table S3). Then, we conducted univariate Cox regression and Kaplan‒Meier analysis and found that six genes (DLAT, DLD, GLS, LIAS, LIPT1 and PDHA1) were significantly correlated with OS in 437 patients with PAAD (Additional file 1: Fig. S6 and Additional file 2: Table S4). In addition, Spearman correlation analysis revealed the correlation of these six genes (Additional file 1: Fig. S7A). Next, the comprehensive landscape of CRG interactions, regulator connections, and their prognostic value in patients with PAAD was demonstrated in a cuproptosis network (Fig. 3A). To further explore the classification of cuproptosis in PAAD, we used an unsupervised clustering analysis to classify the PAAD patients based on the expression profiles of the six prognostic CRGs. Our results showed that k = 3 appeared to be an optimal selection for categorizing the entire cohort into three subtypes, designated cuproptosis Clusters A-C, respectively (Additional file 1: Fig. S7B–F). Cluster A included 163 cases, Cluster B included 133 cases, and Cluster C included 141 cases. Survival analysis demonstrated that prognosis differed significantly among the three cuproptosis subtypes, and Cluster C had considerable survival advantages (log-rank test, P = 0.041, Fig. 3B). Principal component analysis (PCA) showed that the three clusters could be significantly separated based on the expression levels of CRGs (Fig. 3C). As expected, there were significant differences in the expression of the CRGs among the three subtypes (Fig. 3D). In addition, since the GSE21501 cohort has a large sample size among the four GEO datasets, it was used to validate the repeatability of the clustering. Similarly, three distinct subtypes were categorized using a consensus clustering algorithm in this cohort (Additional file 1: Fig. S8). The Kaplan‒Meier curve showed significant differences among the three clusters (log-rank test, P = 0.025, Fig. 3E), further confirming that there are three cuproptosis clusters in PAAD.

### Characteristics of biological function and TME in the cuproptosis subtypes

**Fig. 4:**
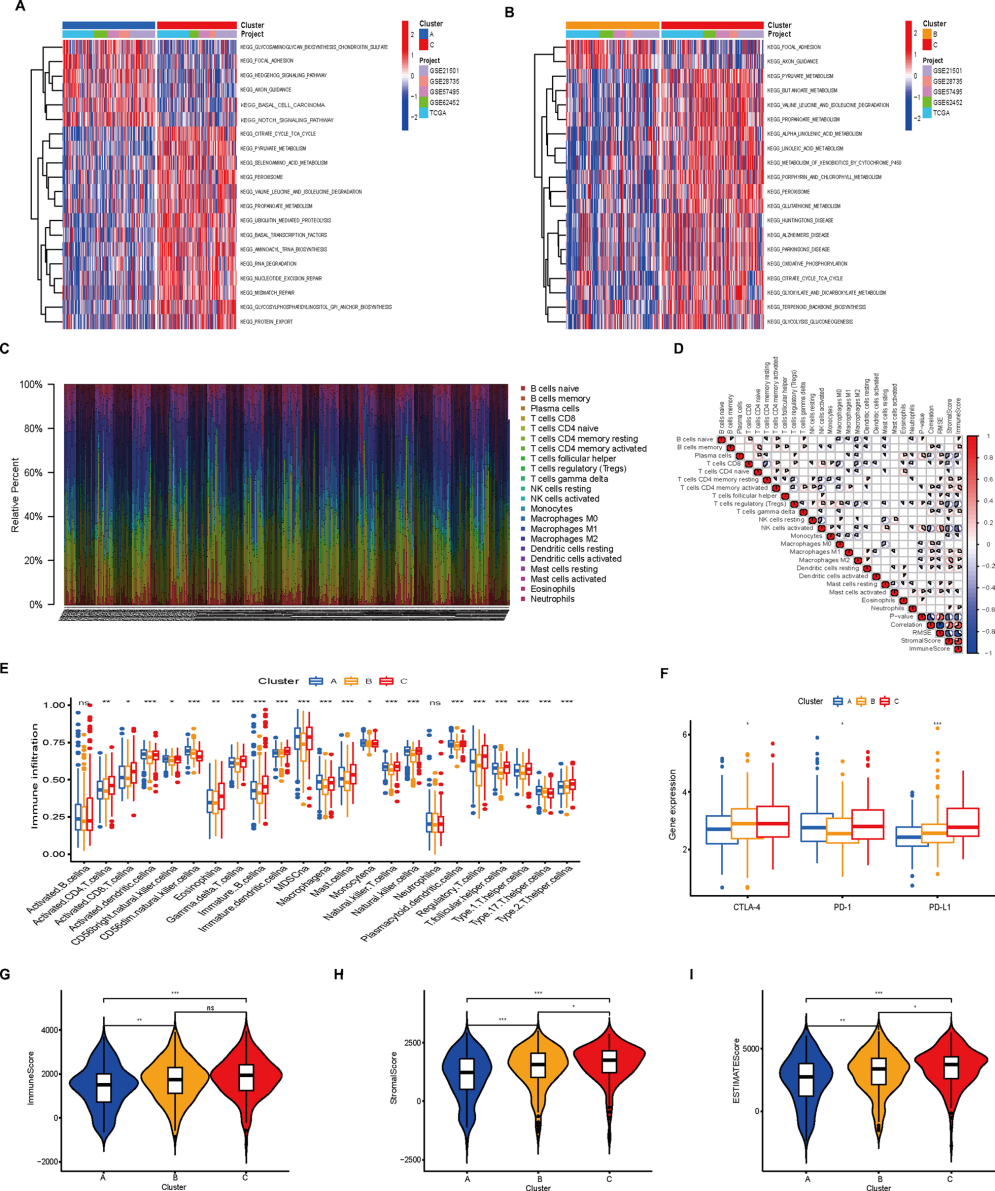
Biological characteristics and TME cell infiltration in cuproptosis subtypes. **A**, **B** GSVA of biological pathways among the three cuproptosis subtypes, in which red and blue represent activated and inhibited pathways, respectively. **A** Cluster A vs. Cluster C; **B** Cluster B vs. Cluster C. **C** Relative proportions of immune infiltration in all PAAD cohorts using the CIBERSORT algorithm. **D** A correlation coefficient heatmap was generated to show the immune cell interaction (the color from blue to red represents negative and positive correlations, and the size of the pie graph represents the absolute correlation coefficient). **E** Abundance of 22 infiltrating immune cell types among the three cuproptosis subtypes. **F** Differences in the expression levels of CTLA-4, PD-1 and PD-L1 among the three subtypes. **G–I** Correlations between the three subtypes and TME score. *P < 0.05, **P < 0.01, ***P < 0.001

To further investigate the difference in survival among the three cuproptosis subtypes, we first conducted GSVA enrichment analysis to assess their functional and biological differences (Fig. 4A, B). The results suggested that Cluster A was mainly enriched in some cancer-related pathways, such as focal adhesion, basal cell carcinoma, and Notch and Hedgehog signaling pathways; Cluster B was involved in focal adhesion and axon guidance pathways; and Cluster C was mainly enriched in tricarboxylic acid (TCA) cycle-related pathways, such as the citric acid cycle, pyruvate metabolism and fatty acid metabolism, as well as some activation repair biological processes, including mismatch repair, nucleotide excision repair and base excision repair. Next, differences in the infiltration of 22 types of immune cells among patients with PAAD are shown in Fig. 4C, suggesting that this is an intrinsic feature reflecting individual differences. Next, a correlation coefficient heatmap provided an intuitive understanding of the state of immune cell interactions of 22 immune cell types (Fig. 4D). To further assess the differences in the infiltration of immune cells among the three clusters, the enrichment score of 22 immune cell types was calculated in the three subtypes using ssGSEA (Fig. 4E). In Cluster A, the most significant immunoinfiltrating cells were activated dendritic cells, natural killer cells, myeloid-derived suppressor cells, monocytes, plasmacytoid dendritic cells, and T helper cells 17. Activated CD4 T cells, activated CD8 T cells, eosinophils, gamma delta T cells, immature B cells, macrophages, natural killer T cells and follicular helper T cells showed the greatest infiltration in Cluster C. The infiltration of immune cells in Cluster B was similar to that in Cluster A. To predict the response to immune checkpoint inhibitors (ICIs), we analyzed the relationship between the expression levels of immune checkpoint genes in different cuproptosis subgroups. As shown in Fig. 4F, the expression of crucial immune checkpoint-related molecules, such as CTLA-4, PD-1 and PD-L1, showed significantly higher expression in subtype C. Next, we examined the TME score (stromal score, immune score and estimate score) of the three clusters, and the result showed that Cluster C had the highest estimate score (Fig. 4G–I). These results suggest a potential correlation between cuproptosiss and the efficacy of immunotherapy.

### Comprehensive analysis of cuproptosis DEGs in PAAD

**Fig. 5:**
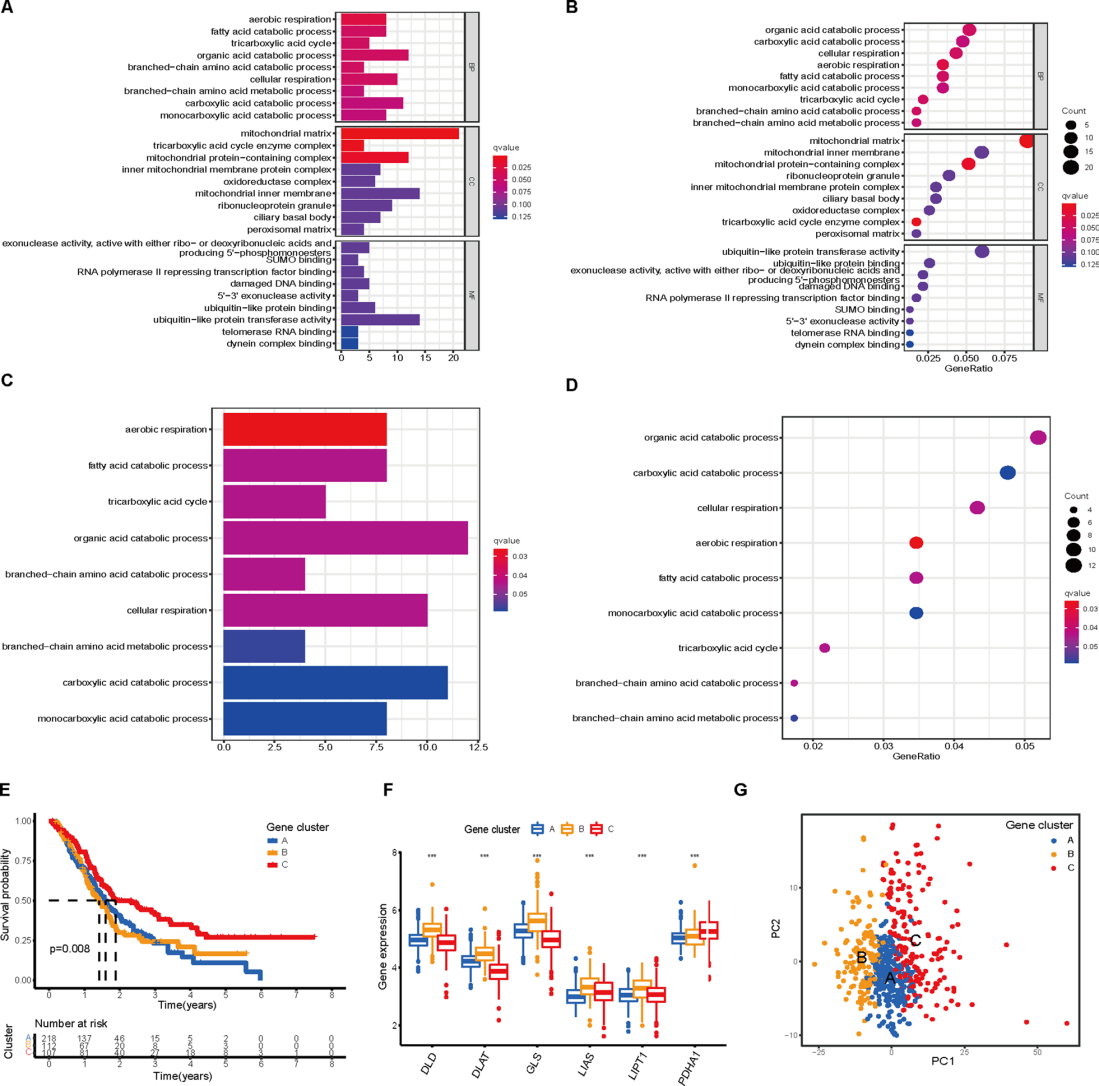
Identification of gene clusters based on DEGs. **A–D** GO and KEGG enrichment analyses of DEGs among the three cuproptosis clusters. **E** Kaplan–Meier curves of the three gene clusters. **F** Differences in the expression levels of CRGs among gene subtypes (***P < 0.001). **G** PCA of DEGs identified three distinct subtypes

To explore the potential function of the cuproptosis subtypes in PAAD, we identified 240 DEGs among the three subtypes (Additional file 1: Fig. S9A). Next, GO functional enrichment analysis revealed that the DEGs were considerably enriched in TCA cycle-related and mitochondrial metabolism biological processes, such as the organic acid catabolic process, carboxylic acid catabolic process, fatty acid catabolic process, tricarboxylic acid cycle, aerobic respiration and mitochondrial matrix (Fig. 5A, B). KEGG also showed that the DEGs were enriched in TCA cycle-related and mitochondrial respiration pathways (Fig. 5C, D). These enrichment analyses indicated that these DEGs are closely associated with the TCA cycle and cellular respiration-related biological processes, which suggests that cuproptosis is linked to mitochondrial metabolism. We then performed univariate Cox regression analysis to examine the prognostic value of the DEGs and screened 40 genes that were significantly associated with OS (P < 0.01), which were used in the subsequent analysis (Additional file 2: Table S5). The protein‒protein network (PPI) of the 40 prognostic DEGs is shown in Additional file 1: Fig. S9B. To further explore the regulatory mechanism, clustering analysis of the 40 DEGs was carried out. The results were similar to the phenotypic clustering of cuproptosis, and three subtypes were identified, namely, gene Clusters A-C (Additional file 1: Fig. S9C-G). Kaplan‒Meier analysis showed that patients in gene Cluster B had the worst OS, while patients in gene Cluster C showed a favorable OS (log-rank test, P = 0.018, Fig. 5E). The three gene clusters showed significant differences in CRG expression, consistent with the expected results of the cuproptosis patterns (Fig. 5F). Moreover, the three clusters could be clearly separated based on the CRGs (Fig. 5G).

### Construction of a prognostic cuproptosis signature

**Fig. 6:**
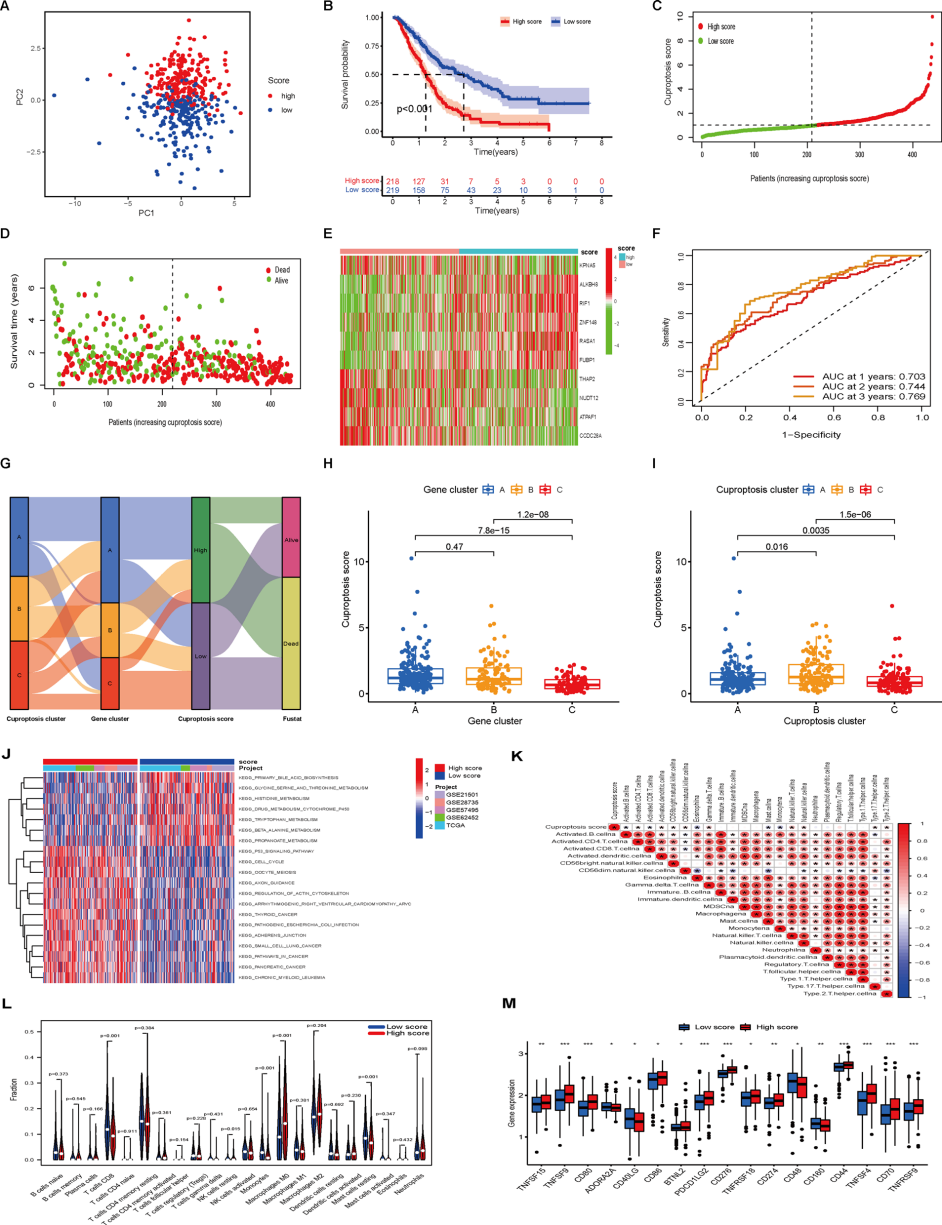
Construction of cuproptosis signature and the biological characteristics and immune status of the signature. **A** PCA between the high and low cuproptosis score groups. **B** Kaplan‒Meier analysis between the two groups. **C–E** Correlation between the prognostic signature and survival outcome of PAAD patients. Distribution of the cuproptosis score (**C**), survival outcome (**D**) and signature gene expression levels (**E**). **F** ROC curve for the survival prediction model. **G** Alluvial diagram displaying the changes in cuproptosis clusters, gene clusters, cuproptosis scores and survival outcomes. **H** Differences in the cuproptosis score between gene clusters. **I** Differences in the cuproptosis score between cuproptosis clusters. **J** GSVA between the two score groups. **K** The correlation between immune cell infiltration and cuproptosis score based on ssGSEA results. **L** Violin plot of immune-infiltrating cells between the two groups. **M** Expression of immune checkpoint genes between the two groups. *P < 0.05, **P < 0.01, ***P < 0.001

To construct a risk signature, these 40 prognostic DEGs were screened by the LASSO regression algorithm (Additional file 1: Fig. S10A, B). As a result, 19 genes were identified in the analysis. Furthermore, 10 genes were identified by multivariate Cox proportional hazards regression analysis, and these genes were further used to construct a prognostic signature (Additional file 1: Fig. S10C), which was defined as the cuproptosis score (Additional file 2: Table S6). Then, PAAD patients were stratified into a high cuproptosis score group and a low cuproptosis score group according to the median cutoff value. The classification ability of the prognostic signature was assessed by PCA (Fig. 6A). As shown in Fig. 6B, the Kaplan‒Meier curve revealed that patients in the high cuproptosis score group had a clearly worse OS than their low score counterparts (log-rank test, P < 0.001). Consistently, patients with high cuproptosis scores had a higher probability of death earlier than those with low cuproptosis scores (Fig. 6C–E). Furthermore, the predictive accuracy of the model was investigated by ROC analysis, with AUC values for 1-, 3-, and 5-year OS of 0.703, 0.744, and 0.769, respectively (Fig. 6F). The distributions of patients in the three cuproptosis clusters, three gene clusters, and two cuproptosis score groups are shown in Fig. 6G. We observed a significant difference in the cuproptosis score in different gene clusters. The cuproptosis score of gene Cluster C was the lowest, while that of gene Clusters A and B showed no significant differences (Fig. 6H). Furthermore, cuproptosis Cluster C had the lowest cuproptosis score compared to cuproptosis Clusters A and B (Fig. 6I). Since cuproptosis Cluster C had the highest immune cell infiltration, these results suggest that a low cuproptosis score may be closely correlated with immune infiltration and that the cuproptosis score may be helpful in predicting cuproptosis clusters in PAAD.

GSVA indicated that the high cuproptosis score group was mainly enriched in some carcinogenic pathways, such as the P53, small cell lung cancer, thyroid cancer, pancreatic cancer and pathways in cancer signaling pathways. Some amino acid and mitochondrial metabolism pathways, including glycine, serine and threonine metabolism and cytochrome P450 pathways, were mainly enriched in PAAD patients with low cuproptosis scores (Fig. 6J and Additional file 2: Table S7). Spearman correlation analysis showed that the cuproptosis score was negatively correlated with activated B cells, CD8 T cells, eosinophils, mast cells and monocytes but positively associated with activated dendritic cells, natural killer cells, Th17 cells and Th2 cells (Fig. 6K). Similarly, the CIBERSORT algorithm revealed that CD8 T cells, monocyte and NK resting cells were higher in the low score group (Fig. 6L). Given the importance of ICIs, a difference was further found in the expression of checkpoint molecules between the two groups (Fig. 6M). The results showed that 17 immune checkpoint genes were differentially expressed between the two score groups, indicating that the cuproptosis score may be a candidate biomarker for checkpoint-based immunotherapy.

### Characteristics of the cuproptosis signature in the TCGA cohort

**Fig. 7:**
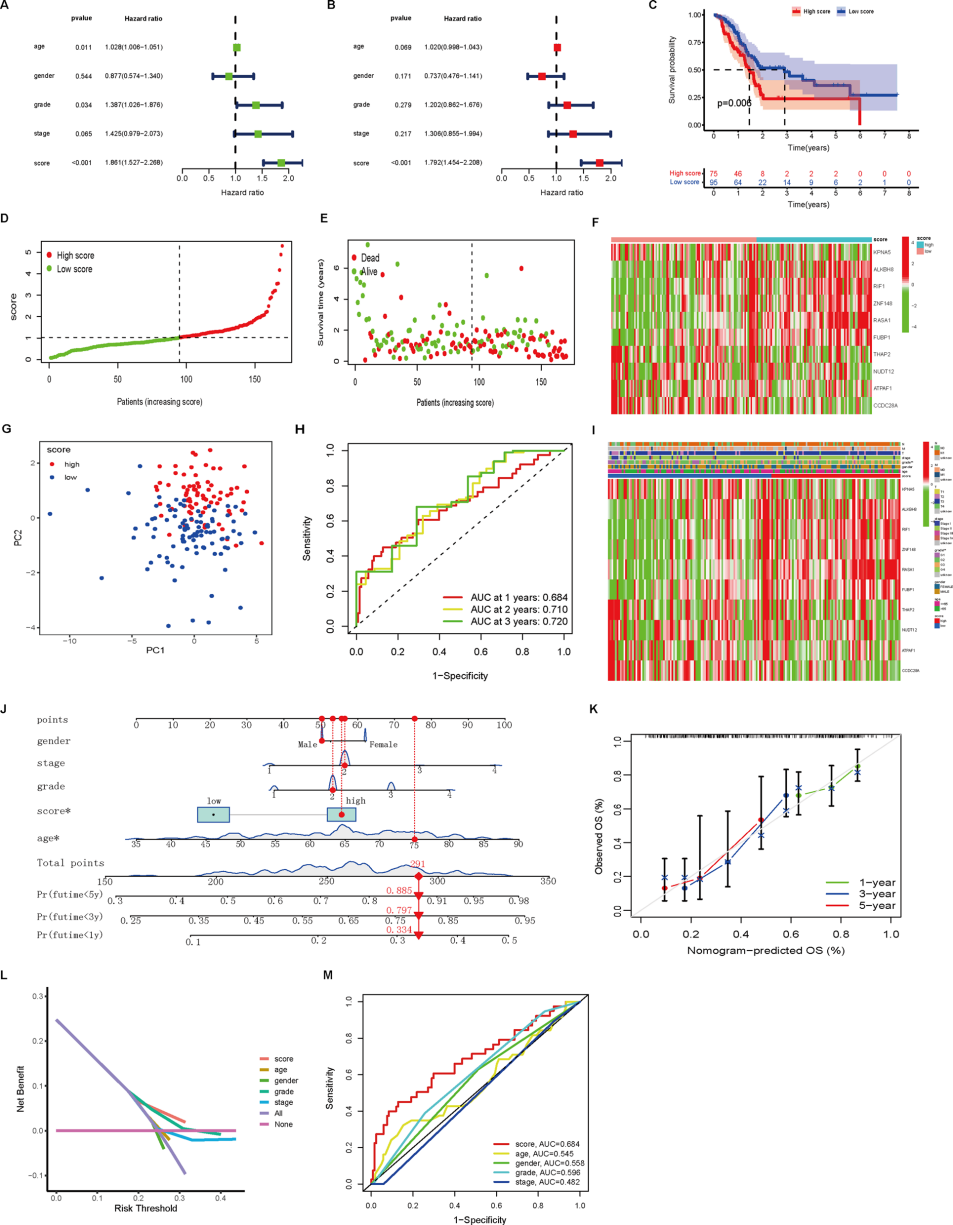
Characteristics of the cuproptosis score in the TCGA cohort. **A**, **B** Forest plots for univariate (A) and multivariate (B) Cox regression analysis of associations between clinical parameters (including cuproptosis score) and OS. **C** Kaplan‒Meier curves of OS between the high and low cuproptosis score groups. **D–F** Correlation between the prognostic signature and survival status of PAAD patients. Distribution of cuproptosis scores (**D**), survival status (**E**) and signature gene expression levels (**F**). **G** PCA between the high score and low score groups. (**H**) ROC curve for the survival prediction model. **I** The heatmap shows the distribution of cuproptosis score genes and clinicopathological factors in the two score groups. **J** A clinical prognostic nomogram was developed to predict 1-, 3-, and 5-year survival. **K** Calibration curves showing nomogram predictions for 1-year, 3-year, and 5-year survival. **L** Decision curve analysis (DCA) of the clinical utility of the nomogram for prognosis prediction. **M** Time-dependent ROC curve analyses for predicting OS at 5 years by cuproptosis score, age, sex, grade and stage

First, univariate Cox regression showed that the cuproptosis score was associated with the OS of PAAD patients (P < 0.001; Fig. 7A). Multivariate Cox regression showed that the cuproptosis score could be used as an independent prognostic factor (HR = 1.792, P < 0.001; Fig. 7B). Then, the difference in survival rate between patients with high and low cuproptosis scores was clearly significant (log-rank test, P = 0.006, Fig. 7C). In addition, a cuproptosis score distribution dot plot showed that there were more surviving patients in the low cuproptosis score group (Fig. 7D–F). The classification ability of the cuproptosis signature was confirmed by PCA (Fig. 7G). We next evaluated the predictive sensitivity and specificity of the cuproptosis score by ROC curves. The AUCs at 1, 2, and 3 years reached 0.684, 0.710, and 0.720, respectively (Fig. 7H). Furthermore, the correlations between the prognostic signature and clinicopathological factors are shown as a heatmap (Fig. 7I). To examine the potential clinical practicality of the prognostic signature, we constructed a nomogram with the cuproptosis score and clinicopathological factors to estimate the 1-, 3-, and 5-year survival probabilities of patients with PAAD (Fig. 7J). The calibration curve of the nomogram indicated that the predicted OS rate was close to the actual OS rate at 1, 3, and 5 years (Fig. 7K). Furthermore, decision curve analysis (DCA) revealed that the nomogram based on the cuproptosis score had better clinical practicality for the prognosis prediction of PAAD patients (Fig. 7L). Finally, we generated a 5-year OS time-dependent ROC curve. The AUC value of the clinical prognostic nomogram was 0.684, which was significantly higher than that of age, sex, grade and stage (Fig. 7M), further implying the discriminative ability of the cuproptosis score combined with pathological characteristics to predict the OS of patients with PAAD.

### Evaluation of pathway enrichment, immune infiltration and TMB between the two cuproptosis score groups

**Fig. 8:**
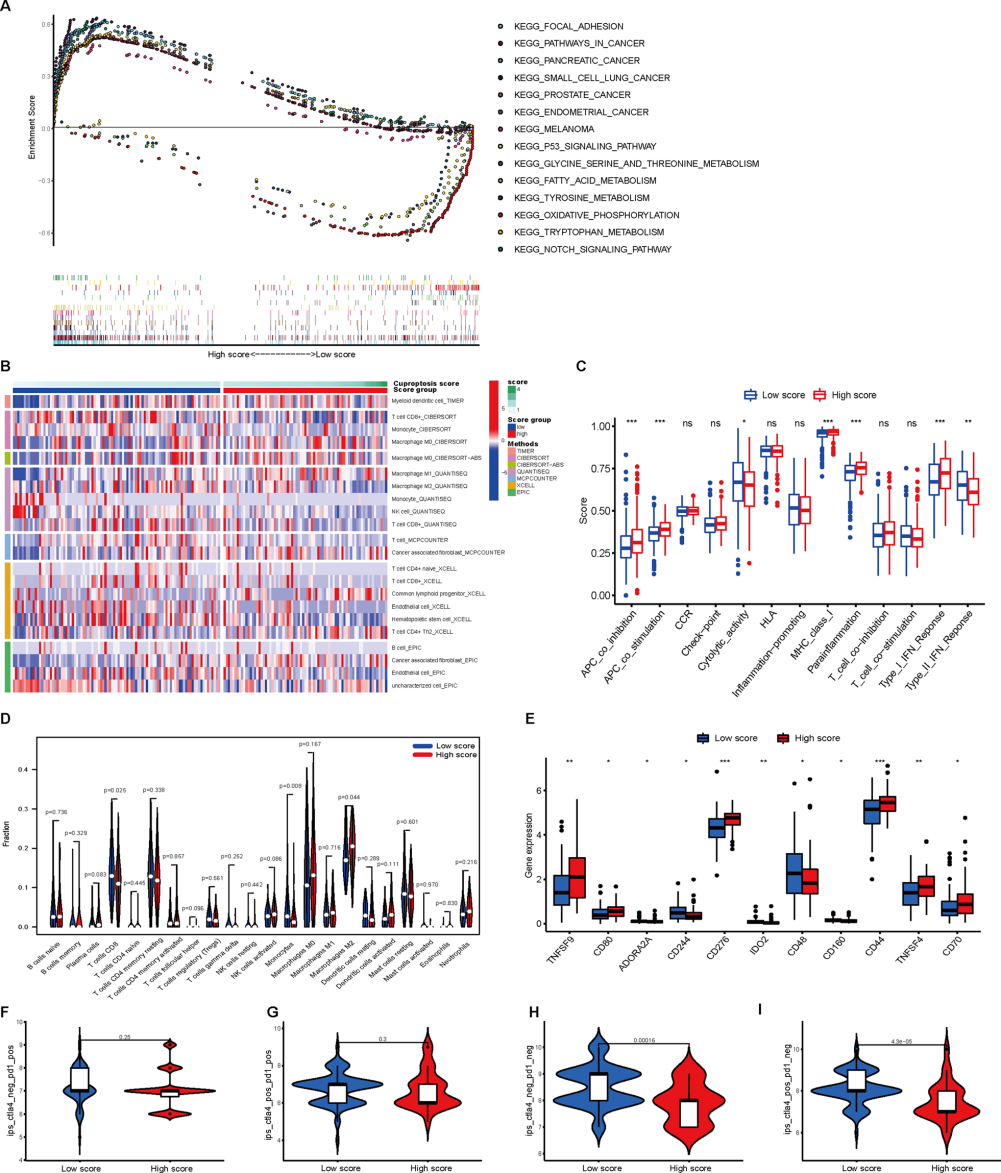
Correlations of the cuproptosis score with biological characteristics and immune checkpoints between the two score groups. **A** GSEA results showing differential enrichment of genes in KEGG between the two groups. **B** Heatmap for immune responses based on the TIMER, CIBERSORT, CIBERSORT-ABS, QUANTISEQ, MCP-COUNTER, XCELL and EPIC algorithms in the two groups. **C** Boxplots showing the results for immune function scores using ssGSEA between the two groups. **D** Violin plot of immune cell infiltration between the low and high cuproptosis score groups. **E** Expression of immune checkpoints between the two groups. **F–I** The relationship between the IPS and two cuproptosis score groups in TCGA-PAAD patients. *P < 0.05, **P < 0.01, ***P < 0.001

GSEA revealed that 30 pathways were significantly clustered in the high cuproptosis score group, including focal adhesion, pathways in cancer, small cell lung cancer, thyroid cancer, pancreatic cancer, chronic myeloid leukemia, cell cycle, and Notch and P53 signaling pathways (Fig. 8A and Additional file 2: Table S8, false discovery rate: q < 0.05). These results are consistent with the results obtained from the whole PAAD cohort. Next, a heatmap of immune infiltration using the TIMER, CIBERSORT, CIBERSORT-ABS, QUANTISEQ, MCP-COUNTER, XCELL and EPIC algorithms is shown in Fig. 8B. Comparative analysis of immune functions and immune cells was implemented to evaluate the differences in APC coinhibition, APC costimulation, cytolytic activity, MHC class I, type I IFN response, type II IFN response, CD8 T cells, monocytes and M2 macrophages between the two score groups (P < 0.05, Fig. 8C, D). In addition, 11 immune checkpoint genes were differentially expressed between the two groups (Fig. 8E), suggesting that cuproptosis score was correlated with ICIs. Recent reports have highlighted the role of the Immunophenoscore (IPS) based on tumor immunogenicity in predicting the immunotherapy response to ICI therapy [25]. Here, we utilized IPS values to analyze the correlation between the cuproptosis signature and immune response. The results showed that there was no significant difference in IPS-PD1/PD-L1/PD-L2 or IPS-PD1/PD-L1/PD-L2 + CTLA4 between the high and low cuproptosis score groups (Fig. 8F, G). Nevertheless, the IPS and IPS-CTLA4 were significantly higher in the low cuproptosis score group, indicating that patients in the low score group might respond better to immunotherapy (Fig. 8H, I).

**Fig. 9:**
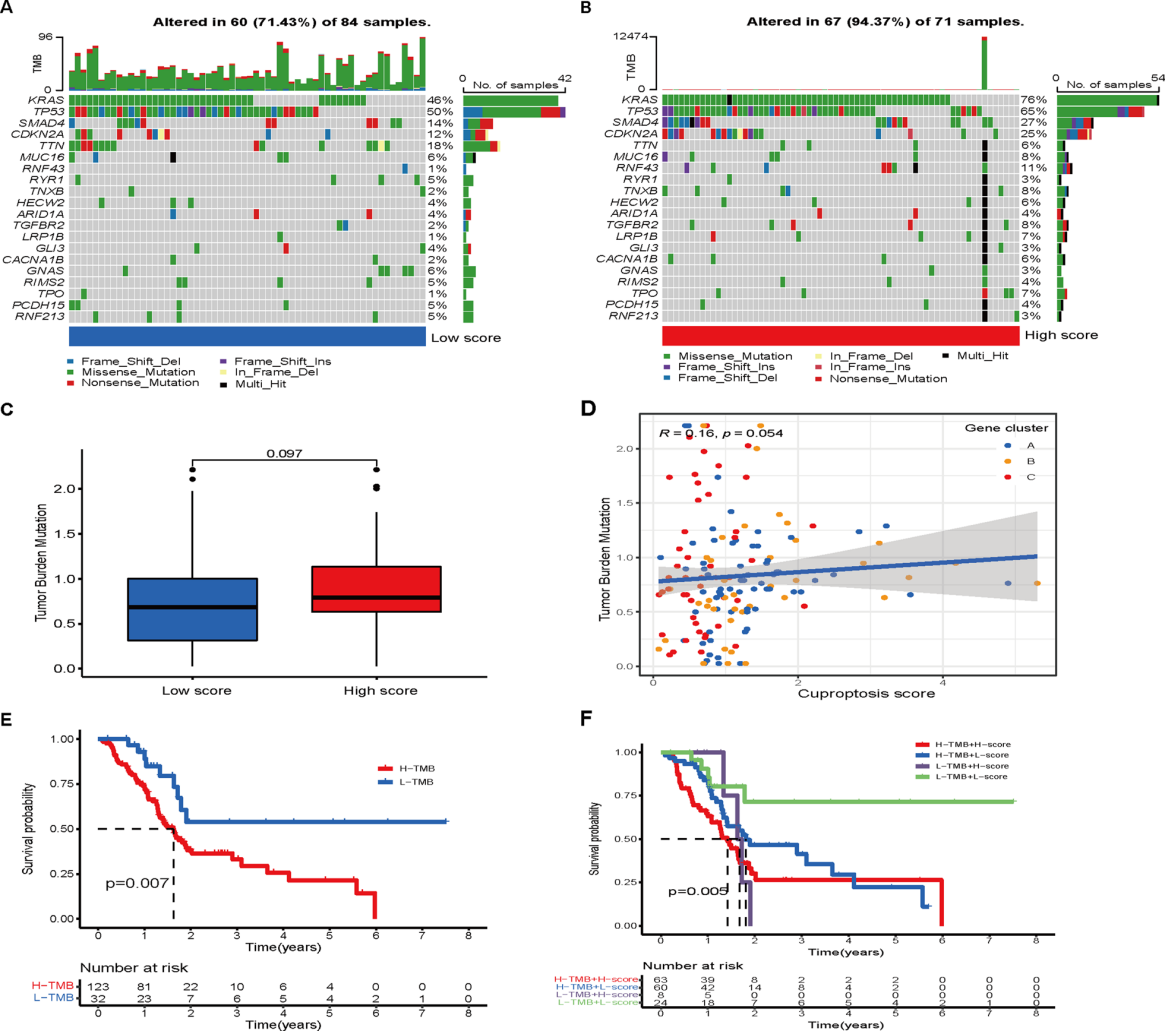
Mutation analysis between the two score groups. **A**, **B** Waterfall plot showing the differences in the tumor somatic mutation landscape between the two score groups. **C** TMB in different cuproptosis score groups. **D** Spearman correlation analysis of the cuproptosis score and TMB. **E**, **F** Kaplan–Meier survival analysis in different cuproptosis scores and TMB groups

In addition, somatic mutations were compared between the two cuproptosis score groups in the TCGA-PAAD cohort, and the top 20 genes with the highest mutation frequency were visualized (Fig. 9A, B). Patients with a high cuproptosis score had noticeably higher frequencies of KRAS, TP53, SMAD4 and CDKN2A mutations than patients with a low cuproptosis score (Fig. 9A, B). However, no significant differences in tumor mutation burden were observed between the two groups (Fig. 9C, D). Survival analysis results showed that OS was significantly worse for patients in the high TMB group than for those in the low TMB group (P = 0.007, Fig. 9E). Furthermore, PAAD patients were divided into four groups for survival analysis based on TMB level and cuproptosis score. A significant difference was found in OS among the four subgroups, and patients in the high TMB and high cuproptosis score groups suffered shorter survival times than those in the low TMB and low cuproptosis score groups (P = 0.005, Fig. 9F). Finally, we evaluated the associations between the cuproptosis score and the efficacy of currently used chemotherapy drugs for PAAD. Intriguingly, we found that the patients in the high cuproptosis score group had significantly lower IC50 values for gemcitabine, paclitaxel, camptothecin and gefitinib (Additional file 1: Fig. S11), suggesting that the cuproptosis score may be associated with drug sensitivity.

### Validation of the expression levels of CRGs in cells and tissues

**Fig. 10:**
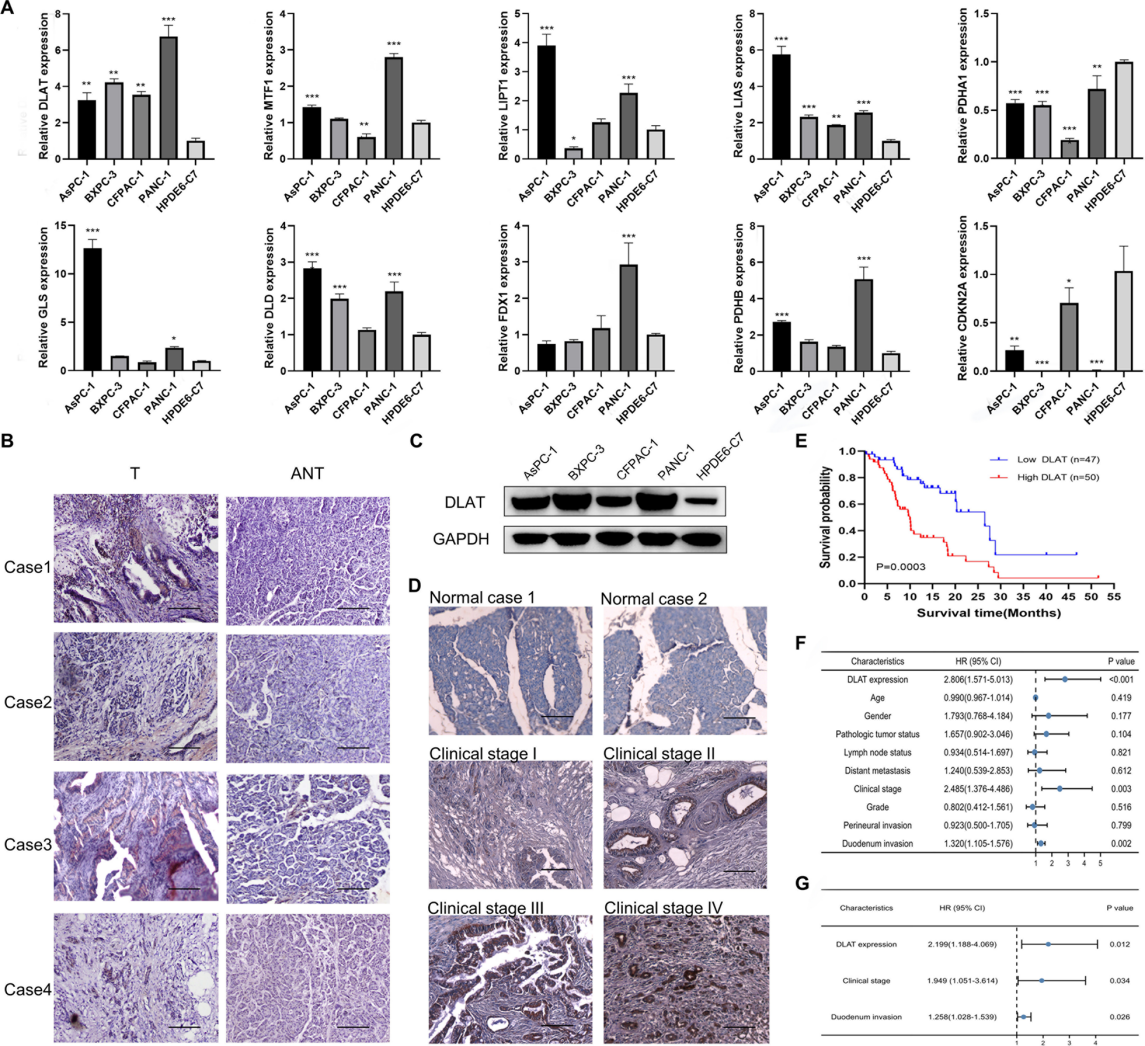
Validation of the expression levels of CRGs in cells and tissues. **A** Comparison of the expression levels of 10 CRGs in pancreatic cancer cell lines and normal cells using qRT‒PCR. *P < 0.05, **P < 0.01, ***P < 0.001. **B** Comparison of the expression levels of DLAT in PAAD tissues and adjacent nontumorous tissues (ANT) via IHC (scale bar: 100 μm). **C** Western blot analysis of DLAT protein levels; GAPDH was used as a loading control. **D** IHC staining indicated that DLAT was overexpressed in PAAD tissues (clinical stages I–IV) compared to in normal pancreatic tissues (scale bar: 100 μm). **E** Kaplan‒Meier curves of PAAD patients with low versus high expression levels of DLAT. **F**, **G** Forest plots for univariate (**F**) and multivariate (**G**) Cox regression analyses of the prognostic value

To verify the reliability of the CRG expression profiles in PAAD obtained from the public database, we examined their relative expression levels in pancreatic cancer cell lines (AsPC-1, BxPC-3, CFPAC-1 and PANC-1) and an immortalized human pancreatic ductal epithelial cell line HPDE6-C7 using qRT‒PCR. As shown in Fig. 10A, the expression levels of most CRGs were relatively upregulated in PAAD cell lines compared with normal controls. Next, the GEPIA database [30] was utilized to acquire the expression of CRGs in PAAD tissues and normal tissues. Similarly, the expression levels of CRGs showed an overall upward trend in PAAD tissues (Additional file 1: Fig. S12A). We further explored the protein expression levels of these CRGs in PAAD using the Human Protein Atlas (HPA) database [31]. Consistent with the above results, the protein levels of FDX1 and CDKN2A were not expressed in normal pancreatic tissues, while medium and high expression levels of these two proteins were observed in PAAD tissues (Additional file 1: Fig. S12B). Low protein expression levels of DLAT, GLS, DLD, LIAS, LIPT1, PDHA1, PDHB and MTF1 were detected in normal pancreatic tissues, whereas medium or high expression levels of these proteins were observed in PAAD tissues (Additional file 1: Fig. S12B). Together, these results suggested that the transcriptional and translational expression levels of the 10 CRGs were overexpressed in PAAD. Next, DLAT, a subunit of the pyruvate dehydrogenase complex, was selected as a candidate gene for further validation of prognostic prediction, as its P value ranked lowest in univariate prognostic analysis (Additional file 2: Table S4). In addition, the expression level and function of DLAT in PAAD have yet to be established. By using an IHC assay, the DLAT protein level was found to be markedly higher in PAAD tissues than in adjacent nontumorous tissues (Fig. 10B). DLAT was primarily localized in the cytoplasm of PAAD cells. In addition, western blot analysis confirmed that the expression of DLAT was obviously increased in pancreatic cancer cells compared to in normal control cells (Fig. 10C and Additional file 1: Fig. S13). We further detected the expression levels of DLAT in 97 paraffin-embedded PAAD samples by IHC staining. The results confirmed that DLAT was overexpressed in PAAD tissues compared to in normal pancreatic tissues (Fig. 10D). Clinicopathological analyses showed that DLAT expression was significantly correlated with clinical stage and duodenal invasion (Additional file 2: Table S9). Survival analysis revealed that patients with higher DLAT expression levels had clearly lower 5-year OS rates (log-rank test, P = 0.0003, Fig. 10E). Furthermore, multivariate Cox regression analysis showed that high DLAT expression was an independent poor prognostic factor (HR, 2.199; P = 0.012, Fig. 10F, G, Additional file 2: Table S10). These results suggest that upregulation of DLAT might promote the progression of PAAD.

## Discussion

Due to the high level of heterogeneity in PAAD, the prognosis of patients with PAAD is considerably poor. Cuproptosis is a new form of programmed cell death mediated by a series of CRGs; we comprehensively investigated the integrated roles of these CRGs in the molecular typing of PAAD and their relationship with the TME and clinical characteristics. In this study, global alterations in CRGs at the transcriptional and genetic levels in PAAD were evaluated. We integrated the genomic profiling of the CRGs and identified three distinct cuproptosis subtypes in 437 PAAD samples from five cohorts. Survival analysis showed that subtype C had significant survival advantages. Analysis of TME cell infiltration showed a high level of heterogeneity among these three subtypes. Finally, we constructed a cuproptosis signature to further evaluate the cuproptosis subtypes and their relationship with immune infiltration.

To the best of our knowledge, there has been no systematic study of CRGs in different human cancers to date or the correlations between CRGs and the development of PAAD. Here, we first utilized TCGA pan-cancer data to systematically analyze the 10 CRGs in 33 cancer types. The expression and survival analysis showed strong heterogeneity of CRGs across different cancer types. Then, immune infiltrate subtype and TME analyses confirmed that these CRGs were involved in the immune response and TME. Additionally, we found that the expression levels of the CRGs were closely related to cancer stemness, providing novel insights into cuproptosis. Finally, drug sensitivity analysis proved that cuproptosis was associated with chemotherapy resistance. Together, our results will greatly help in elucidating the role of CRGs in tumorigenesis, the TME and drug resistance.

Cuproptosis is a novel type of cell death that is highly linked to mitochondrial metabolism. Tsvetkov et al. demonstrated that intracellular copper accumulation can trigger aggregation of lipoylated mitochondrial enzymes, and copper toxicity was closely associated with pivotal components of the TCA cycle [21]. In the current study, we evaluated the expression of 10 CRGs and the potential value of these genes as prognostic markers for PAAD. All CRGs were differentially expressed between tumors and normal tissues, and six genes were associated with OS prognosis. These results suggested the critical role of CRGs in the progression of PAAD. To test this hypothesis, PAAD samples were divided into three discrete cuproptosis subtypes based on the CRG expression levels, and multi-layer CRG alterations were associated with patient prognosis. GSVA showed that cuproptosis Cluster A was mainly enriched in some cancer-related pathways, and Cluster C was mainly clustered in some TCA cycle-related metabolism pathways, such as the citric acid cycle, pyruvate metabolism and fatty acid metabolism. Furthermore, GO analysis revealed that the DEGs among different cuproptosis subtypes were substantially enriched in TCA cycle-related and aerobic respiration biological processes. KEGG confirmed that the DEGs were clustered in TCA cycle-related and cellular respiration pathways. These results indicated that the cuproptosis subtypes are highly associated with mitochondrial respiration, which is consistent with the results in the abovementioned study. Interestingly, Tsvetkov et al. also demonstrated that cells relying on mitochondrial respiration are more sensitive to cuproptosis than those relying on anaerobic glycolysis [21]. Thus, cuproptosis provides novel insights to exploit copper-induced mitochondrial toxicity to treat PAAD, and patients in cuproptosis Cluster C with active mitochondrial metabolism may be more vulnerable to cuproptosis. As a key hallmark of cancers, metabolic reprogramming triggers the malignant behavior of PAAD, and this metabolic phenotype is characterized by preferential dependence on glycolysis [32]. PAAD cells elicit metabolic conversion from oxide phosphorylation to glycolysis, which is closely linked to mitochondrial dysfunction [33]. In addition, because glycolysis is critical for PAAD tumorigenesis, metastasis and chemotherapy resistance [33, 34], inhibition of glycolysis would not only enfeeble PAAD cell malignant potential but also make them more vulnerable to cuproptosis.

The most significant contribution of this study is the demonstration of the relationship between cuproptosis subtypes and immune infiltration. The number and proportion of infiltrating cells influence tumor progression and the response to immunotherapy and are closely related to patient prognosis. According to the tumor immunoediting hypothesis, the immune system is a double-edged sword: protecting the host by killing tumor cells and selecting less immunogenic tumor cells by editing its genome [35]. This may lead to a decrease in immunoreactive cells, an increase in immunosuppressive cells and ultimately tumor immune escape from immune destruction. Immunotherapy has had significant efficacy for multiple cancers [36] but has not yet been translated to PAAD. PAAD is prominently resistant to current immunotherapies because of its strongly immunosuppressive TME, which is characterized by typically poor infiltration of effector T cells and prominent myeloid inflammation and comprises immunosuppressive cells, such as regulatory T cells and myeloid-derived suppressor cells. Nevertheless, a small subset of PAAD patients with high levels of tumor-infiltrating effector T cells showed exclusively longer survival [37], indicating the potential of effective cancer immunotherapy in PAAD. Hence, we hypothesized that PAAD samples in different cuproptosis subtypes with distinct prognoses would have different immunotherapeutic responses. In this study, we showed that the characteristics of the TME and the relative abundance of 22 immune cell types differed significantly among these cuproptosis subtypes. Our results revealed that cuproptosis Cluster C with a better OS prognosis had an increased infiltration of antitumor immune components, such as activated CD4 T cells, activated CD8 T cells, gamma delta T cells, eosinophils, immature B cells, macrophages and follicular helper T cells, suggesting an immunoreactive characterization of Cluster C. Nonetheless, subtype C also showed a significantly higher expression of immune checkpoint targets, including CTLA-4, PD-1 and PD-L1, which correlated with the recognition of tumor cells by T cells. These results suggested that the favorable prognosis of Cluster C might not just be due to the high levels of TME immune infiltration; however, other mechanisms, such as activation of repair biological processes and metabolism-related pathways, may also play substantial roles. In addition, the infiltration levels of CD8 T cells were significantly higher in the low cuproptosis score group based on the whole PAAD cohorts or the TCGA-PAAD cohort. CD8 T-cell infiltration in tumors has been found to be a fantastic predictor for the response to ICIs. Studies have demonstrated that the expression of CD8 T cells is associated with the survival time of PAAD patients [38, 39]. In this setting, the low cuproptosis score group of patients with PAAD may benefit greatly from tumor-infiltrating CD8 T cells. Additionally, recent studies revealed that CD8 T cells could induce ferroptosis in tumor cells and that ferroptosis presents a new strategy for anticancer immunotherapy [40, 41]. However, another study revealed that increased CD36 expression could induce lipid peroxidation and ferroptosis in CD8 T cells and impair their antitumor ability [42]. These results provide new insights into the relationship between cuproptosis, TME cell infiltration and PAAD. Nonetheless, whether and how cuproptosis or cuproptosis-inducing drugs affect TME cell infiltration and the role of anticancer immune cells remain unclear. Thus far, very few studies have investigated the potential role of cuproptosis in immune cell infiltration, which needs to be clarified in the future.

Cuproptosis is a novel form of cell death that may provide a new idea for cancer treatment. However, many key issues such as the interconnection between cuproptosis and other forms of regulated cell death and immune infiltration remain unclear. Therefore, the present study explored the role of CRGs in PAAD and the molecular characterization of cuproptosis subtypes, and cuproptosis biomarkers useful in predicting the prognosis of PAAD, which can provide novel insights into the treatment modalities of the disease. Recently, Huang et al. [43] developed a CRG index based on 10 CRGs combined with six well-recognized biomarkers (i.e., KRAS, TP53, SMAD4, BRCA1, BRCA2 and CDKN2A) in PAAD using machine learning procedures. As a result, 13 genes comprise the CRG index, including the six well-established biomarkers and seven CRGs. Further studies showed that the CRG index was correlated with patient prognosis, tumor immunology, molecular subtypes and the efficacy of immunotherapy. Furthermore, the upregulation of DLAT, LIPT1 and LIAS in PAAD were confirmed through qRT‒PCR, Western blot and immunofluorescent staining assays, which were consistent with our results. In the current study, we demonstrated that the transcriptional and translational expression levels of the 10 CRGs were overexpressed in PAAD, and that high DLAT expression was an independent poor prognostic factor. These findings may improve our understanding of CRGs in PAAD and provide new ideas for the assessment of prognosis and the development of more effective immunotherapy strategies.

However, several limitations should be taken into consideration in our study. First, due to few studies about the role of cuproptosis in tumors, the information on CRGs provided by the previous study may not be accurate enough, and some unidentified important CRGs may be missing in the cuproptosis gene sets. Second, a large number of PAAD samples are needed to verify the stability of the phenotyping, and further experimental evidence is needed to investigate the relationship between cuproptosis and immunity. In addition, all samples used in our study were obtained retrospectively, and the cuproptosis score signature would be more reliable if it is verified by large-scale prospective studies.

## Conclusions

This study revealed three cuproptosis subtypes with distinct clinical outcomes in PAAD. We demonstrated that CRGs could drive the heterogeneity of TME immune cell infiltration in PAAD. The cuproptosis score could serve as a promising biomarker for predicting patient prognosis and response to immunotherapy.

## Supplementary Information

**Additional file 1: Fig. S1.** Boxplot showing the difference in CRG expression between primary tumor and adjacent normal tissues in each cancer type.** Fig. S2.** Kaplan–Meier curves of overall survival for CRGs in different cancer types (top 23 ranked by P value). **Fig. S3.** Scatter plots for the association between CRG expression and drug sensitivity (top 9 ranked by P value). **Fig. S4.** (**A**) The relationship between CDKN2A mutation and the expression level of CRGs in TCGA-PAAD. (**B**) The relationship between KRAS mutation and the expression level of CRGs in TCGA-PAAD. **Fig. S5.** Scatter plots showing the association between CRG expression and RNAss, DNAss, stromal score, immune score and ESTIMATE score in PAAD using the Pearson correlation test.** Fig. S6.** Kaplan‒Meier survival curves showing the association between CRG expression and overall survival for PAAD patients (P value was calculated by the log-rank test). **Fig. S7.** Consensus clustering of CRGs in PAAD by the k-means method. (**A**) Spearman correlation analysis of the correlation of the 6 CRGs. (**B–E**) Consensus clustering of 6 CRGs in all PAAD cohorts and consensus matrices for k = 2–5. (**F**) The consensus CDF curves are shown for different k values from 2 to 9. **Fig. S8.** Unsupervised clustering of CRGs and consensus matrix heatmaps for k = 2–5 in the GSE21501 cohort. **Fig. S9.** Identification of gene clusters based on DEGs. (**A**) Venn diagram showing the intersection genes among the three cuproptosis clusters. (**B**) The protein‒protein network (PPI) of DEGs based on the STRING database with a combined score > 0.40. (**C–G**) Consensus clustering of DEGs in PAAD cohorts and consensus matrices for k = 2–5. **Fig. S10.** Construction of cuproptosis signature. (**A**) Profiles of LASSO coefficients. (**B**) LASSO penalized Cox regression analysis. The vertical dashed line is at the optimal log (lambda) value. (**C**) Multivariate Cox regression analysis. **Fig. S11.** Relationships between the cuproptosis score and chemotherapy sensitivity. **Fig. S12.** Comparison of the expression levels of the 10 CRGs in PAAD tissues and normal pancreatic tissues. (**A**) Comparison of the differential expression of CRGs in the GEPIA database. (**B**) Representative IHC images of CRGs in PAAD and normal pancreatic tissues derived from the HPA database. **Fig. S13.** Western blot analysis of DLAT protein levels in pancreatic cancer cell lines and normal cells. (**A**) The related expression levels of DLAT in AsPC-1, BXPC-3, CFPAC-1, PANC-1 and HPDE6-C7 cell lines, respectively. (**B, C**) Western blot analysis of DLAT and GAPDH in the aforementioned cell lines.

**Additional file 2:** **Table S1.** Primers used in this study. **Table S2.** The association between CRG expression and drug sensitivity. **Table S3.** Data set information included in this study for cuproptosis classification. **Table S4.** Univariate Cox regression analysis of CRGs in PAAD patients. **Table S5.** Univariate Cox regression analysis of differentially expressed genes (DEGs) in PAAD patients. **Table S6.** Multivariate Cox regression analysis for the cuproptosis signature based on 10 DEGs. **Table S7.** The activation states of biological pathways between the two cuproptosis score group by GSVA enrichment analysis. **Table S8.** Gene set enrichment KEGG analysis results based on the signature in the TCGA cohort. **Table S9.** Correlations between DLAT expression and clinicopathologic characteristics of PAAD patients. **Table S10.** Univariate and multivariate Cox regression analyses of prognostic variables in PAAD patients.

## Data Availability

The datasets analyzed during the current study are available in the TCGA-PAAD project (http://www.cancer.gov/tcga) and GEO (https://www.ncbi.nlm.nih.gov/geo/query/acc.cgi?acc=GSE62452/GSE28735/GSE21501/57495). TCGA pan-cancer data, including RNA-Seq, clinical data, stemness scores and immune subtypes can be found in UCSC Xena (https://xenabrowser.net/datapages/).

## Abbreviations

PAAD: Pancreatic adenocarcinoma
CRGs: Cuproptosis-related genes
TME: Tumor microenvironment
ICIs: Immune checkpoint inhibitors
ANOVA: Analysis of variance
DNAss: Tumor stemness based on DNA methylation
RNAss: Tumor cell stemness based on mRNA expression
TCGA: The Cancer Genome Atlas
GEO: Gene expression omnibus
GTEx: Genotype-tissue expression database
DEGs: Differentially expressed genes
ROC: Receiver operating characteristic
AUC: Area under the ROC curve
PCA: Principal component analysis
GSVA: Gene set variation analysis
MsigDB: Molecular signatures database
GSEA: Gene set enrichment analysis
IPS: The immunophenoscore
TCIA: The cancer immunome atlas
IHC: Immunohistochemistry
DLAT: Dihydrolipoamide S-acetyltransferase
OS: Overall survival
TMB: Tumor mutation burden
CNV: Copy number variation
ssGSEA: Single-sample gene set enrichment analysis
MAF: Mutation annotation format
