# Supplementary material for: Molecular subtypes based on cuproptosis-related genes and tumor microenvironment infiltration characteristics in pancreatic adenocarcinoma
Source: Cancer Cell Int. 2023 Jan 16;23:7. doi: 10.1186/s12935-022-02836-z (PMC9844034; doi:10.1186/s12935-022-02836-z)
Supplement: Supplementary file 1 — Additional file 1: Fig. S1. Boxplot showing the difference in CRG expression between primary tumor and adjacent normal tissues in each cancer type. Fig. S2. Kaplan–Meier curves of overall survival for CRGs in different cancer types (top 23 ranked by P value). Fig. S3. Scatter plots for the association between CRG expression and drug sensitivity (top 9 ranked by P value). Fig. S4. (A) The relationship between CDKN2A mutation and the expression level of CRGs in TCGA-PAAD. (B) The relationship between KRAS mutation and the expression level of CRGs in TCGA-PAAD. Fig. S5. Scatter plots showing the association between CRG expression and RNAss, DNAss, stromal score, immune score and ESTIMATE score in PAAD using the Pearson correlation test. Fig. S6. Kaplan‒Meier survival curves showing the association between CRG expression and overall survival for PAAD patients (P value was calculated by the log-rank test). Fig. S7. Consensus clustering of CRGs in PAAD by the k-means method. (A) Spearman correlation analysis of the correlation of the 6 CRGs. (B–E) Consensus clustering of 6 CRGs in all PAAD cohorts and consensus matrices for k = 2–5. (F) The consensus CDF curves are shown for different k values from 2 to 9. Fig. S8. Unsupervised clustering of CRGs and consensus matrix heatmaps for k = 2–5 in the GSE21501 cohort. Fig. S9. Identification of gene clusters based on DEGs. (A) Venn diagram showing the intersection genes among the three cuproptosis clusters. (B) The protein‒protein network (PPI) of DEGs based on the STRING database with a combined score > 0.40. (C–G) Consensus clustering of DEGs in PAAD cohorts and consensus matrices for k = 2–5. Fig. S10. Construction of cuproptosis signature. (A) Profiles of LASSO coefficients. (B) LASSO penalized Cox regression analysis. The vertical dashed line is at the optimal log (lambda) value. (C) Multivariate Cox regression analysis. Fig. S11. Relationships between the cuproptosis score and chemotherapy sensitivity. Fig. S12. Comparison [file 12935_2022_2836_MOESM1_ESM.docx]

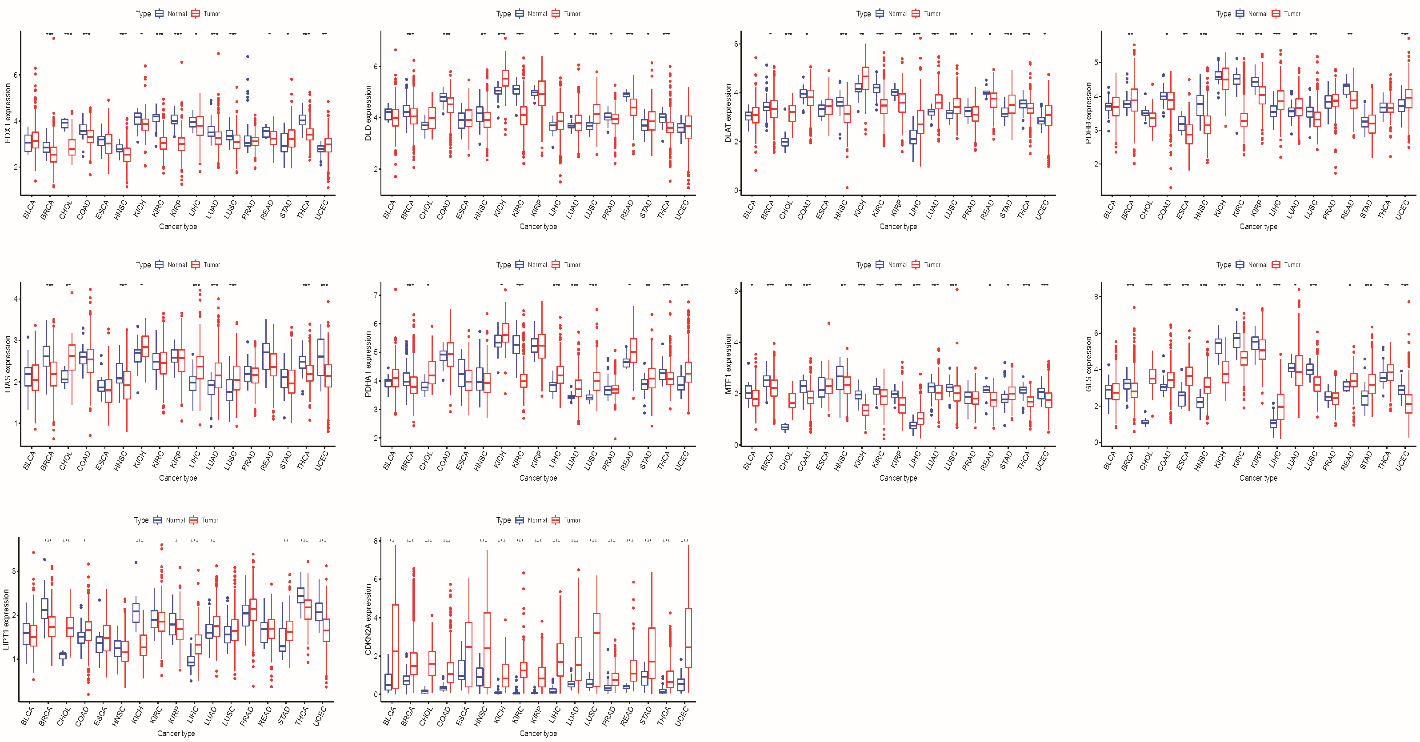


**Fig. S1.** Boxplot showing the difference in CRG expression between primary tumor and adjacent normal tissues in each cancer type.


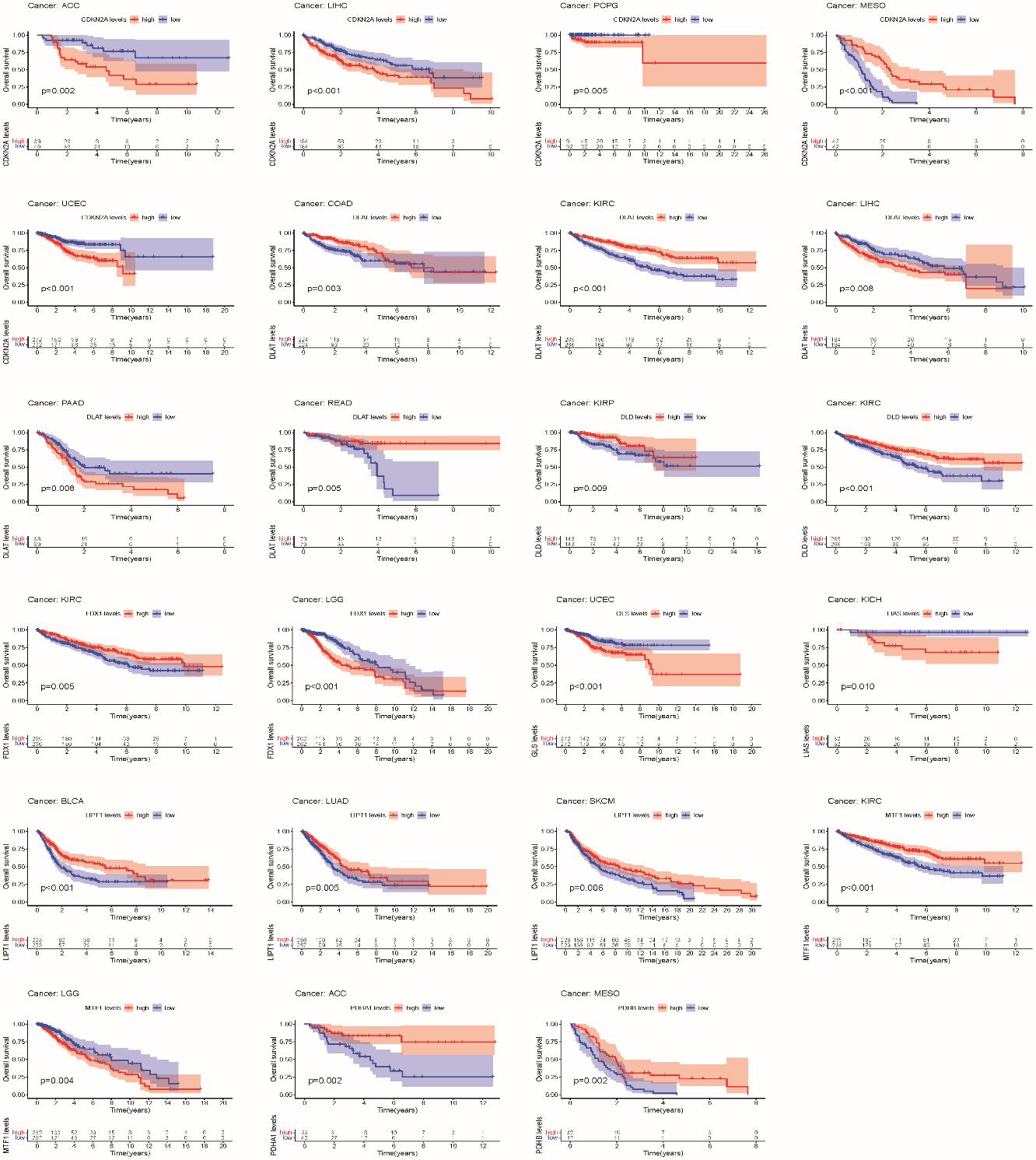


**Fig. S2.** Kaplan–Meier curves of overall survival for CRGs in different cancer types (top 23 ranked by P value).


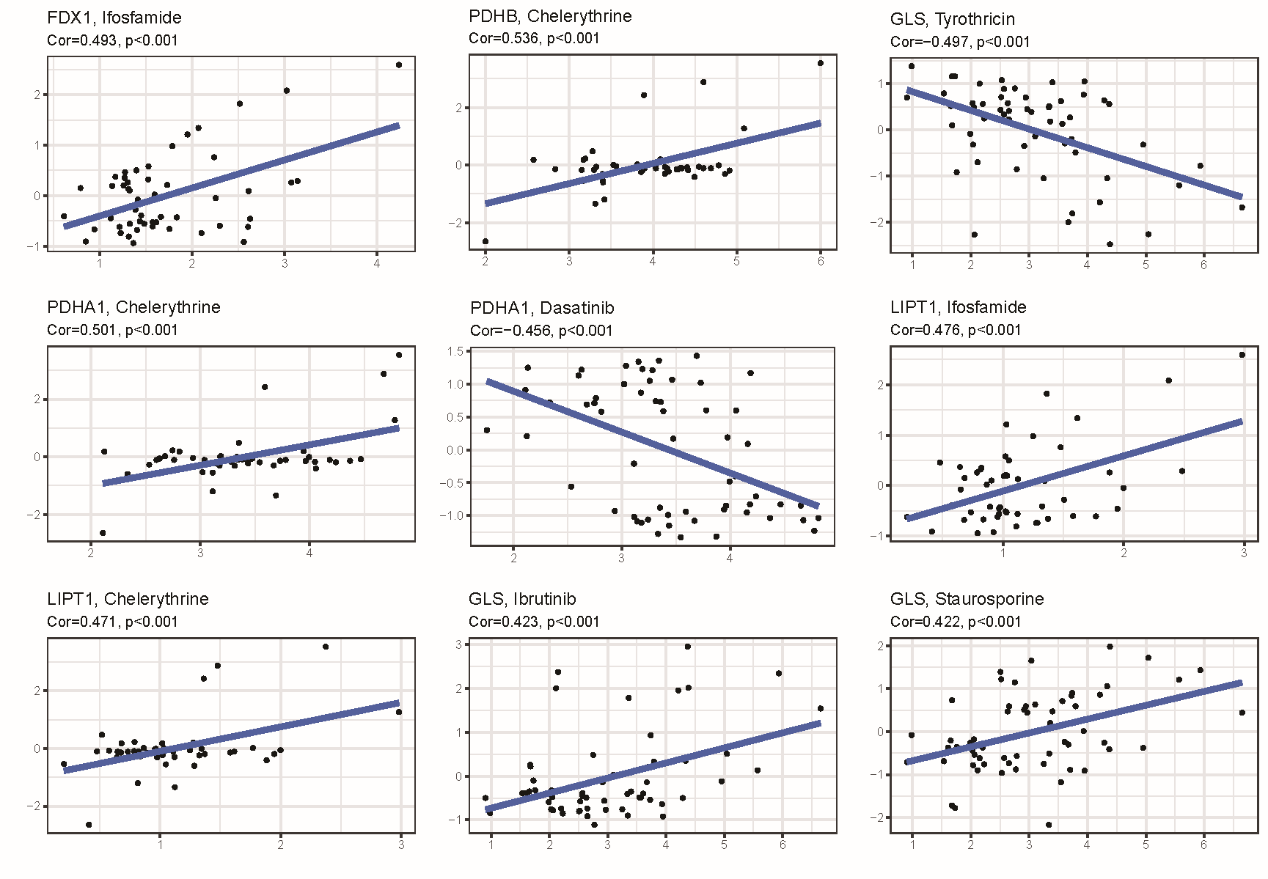


**Fig. S3.** Scatter plots for the association between CRG expression and drug sensitivity (top 9 ranked by P value)


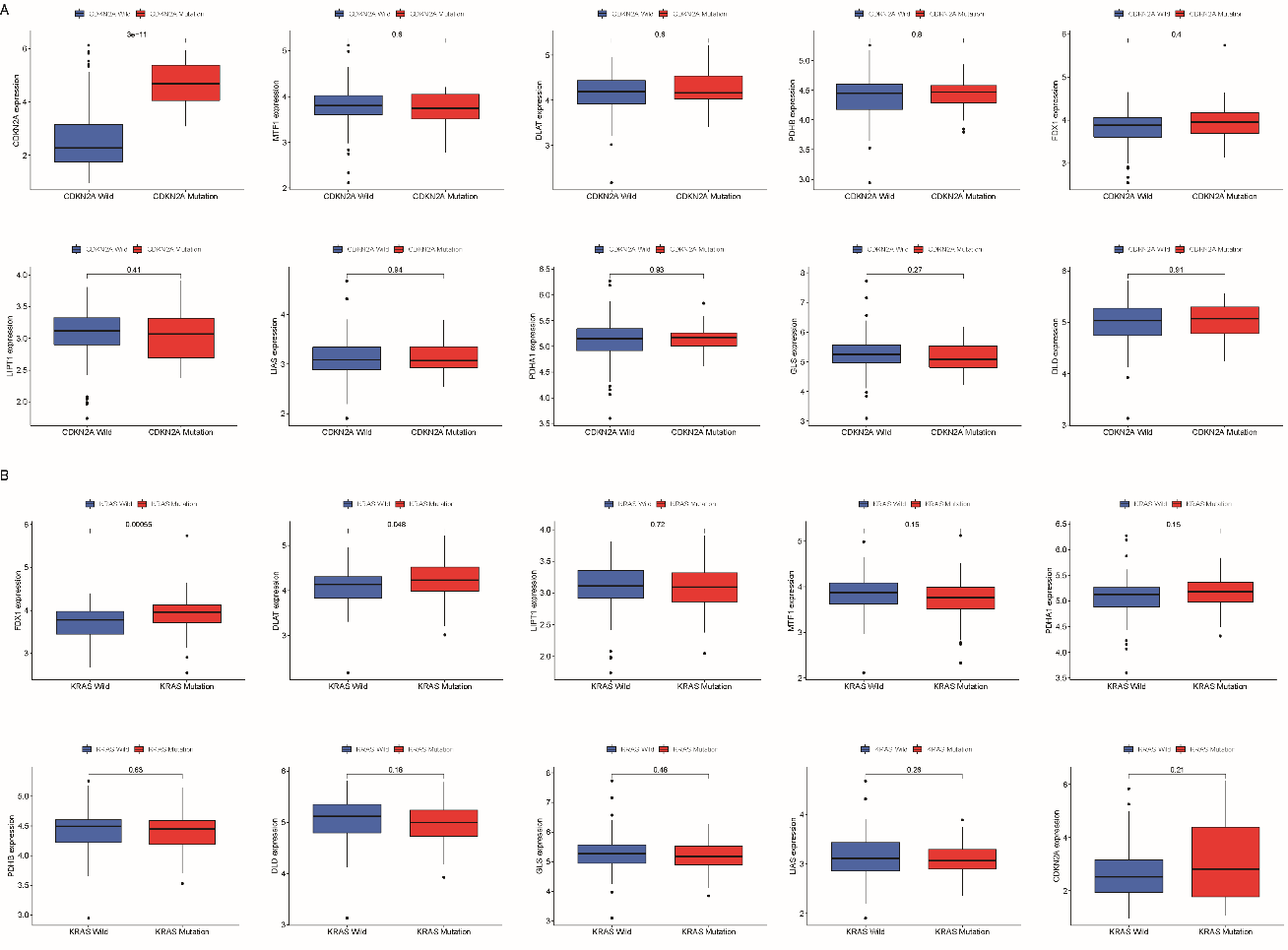


**Fig. S4.** (**a**) The relationship between CDKN2A mutation and the expression level of CRGs in TCGA-PAAD. (**b**) The relationship between KRAS mutation and the expression level of CRGs in TCGA-PAAD.


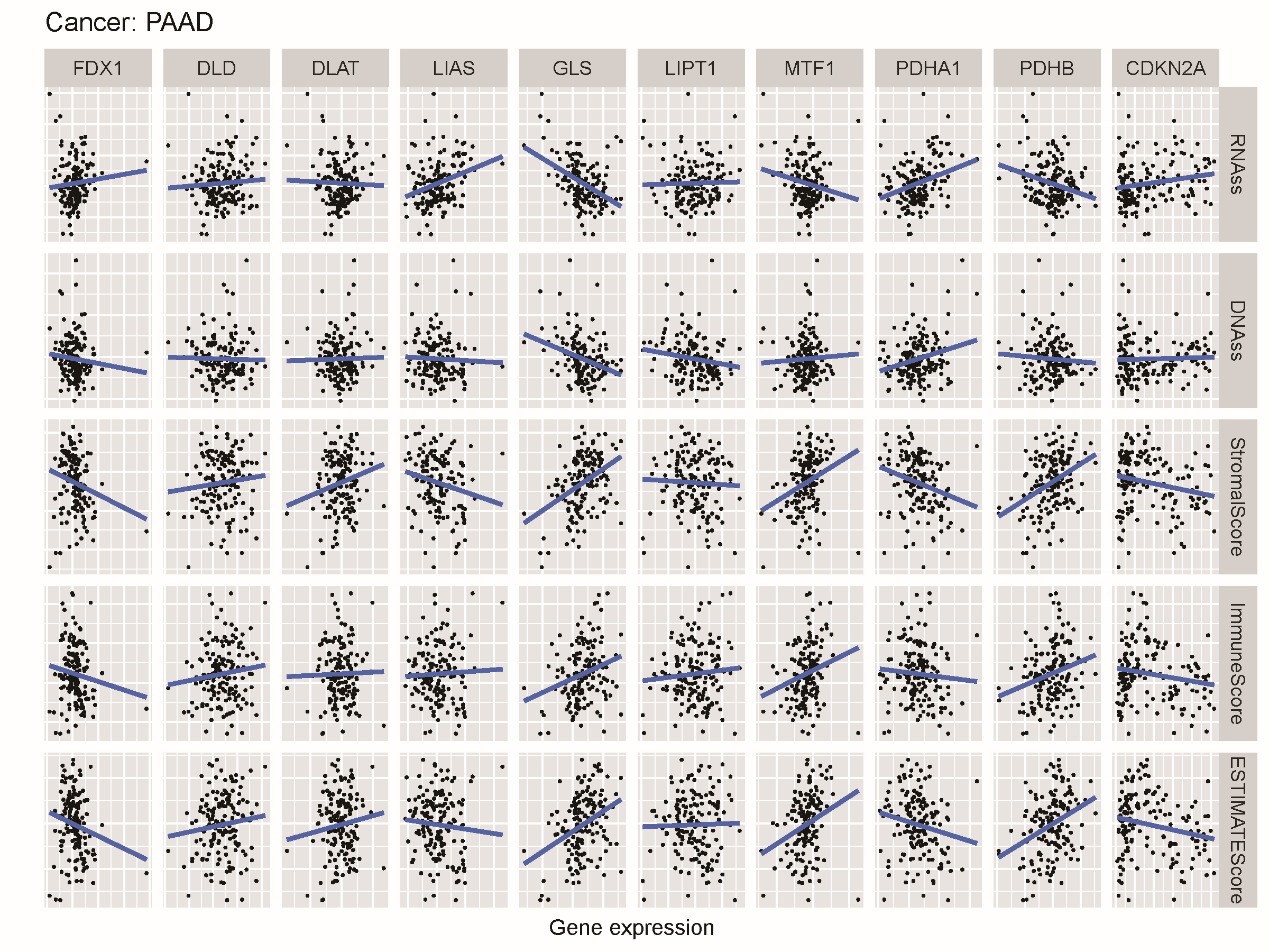


**Fig. S5.** Scatter plots showing the association between CRG expression and RNAss, DNAss, stromal score, immune score and ESTIMATE score in PAAD using the Pearson correlation test.


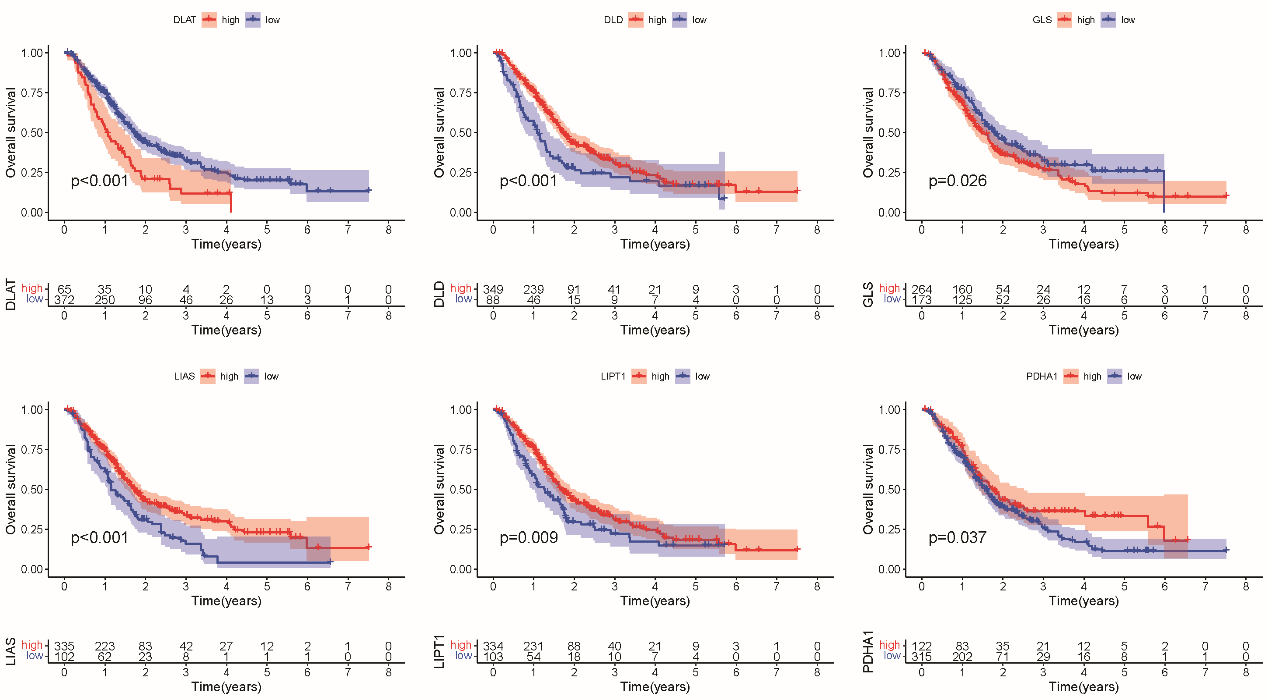


**Fig. S6.** Kaplan‒Meier survival curves showing the association between CRG expression and overall survival for PAAD patients (P value was calculated by the log-rank test).


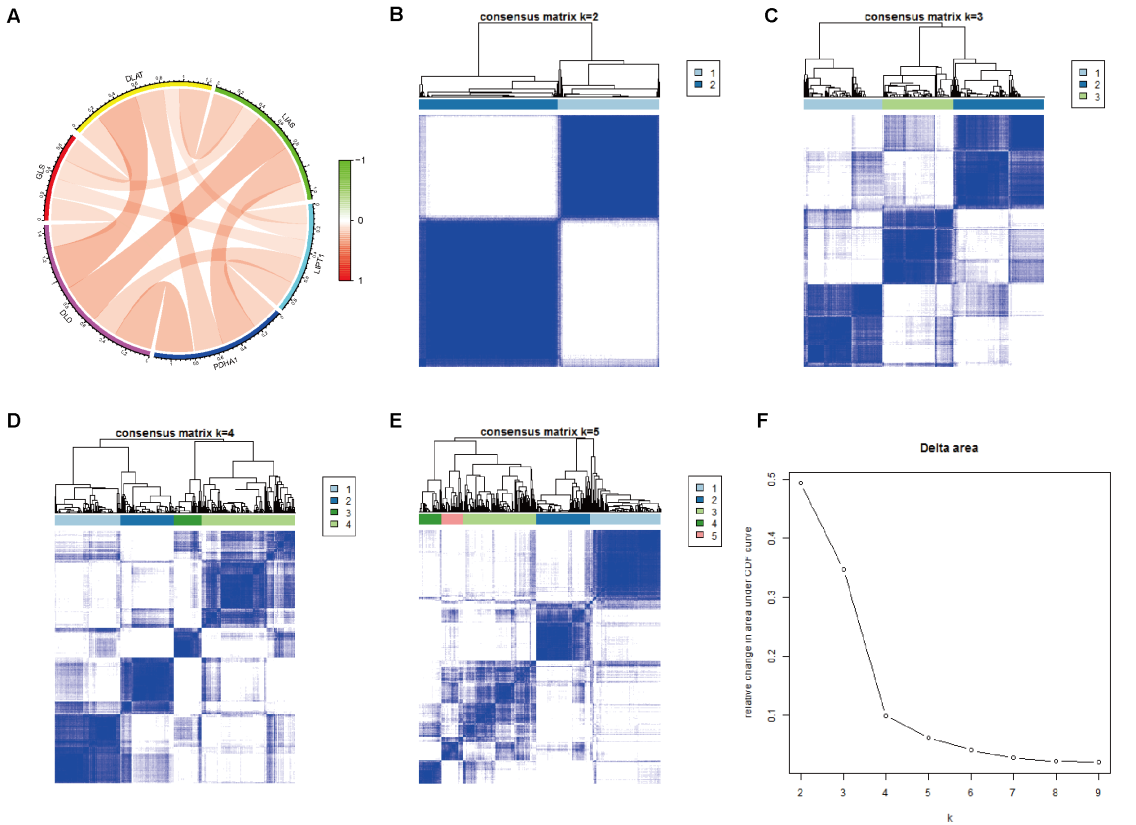


**Fig. S7. Consensus clustering of CRGs in PAAD by the k-means method.** (**A**) Spearman correlation analysis of the correlation of the 6 CRGs. (**B-E**) Consensus clustering of 6 CRGs in all PAAD cohorts and consensus matrices for k=2-5. (**F**) The consensus CDF curves are shown for different k values from 2 to 9.


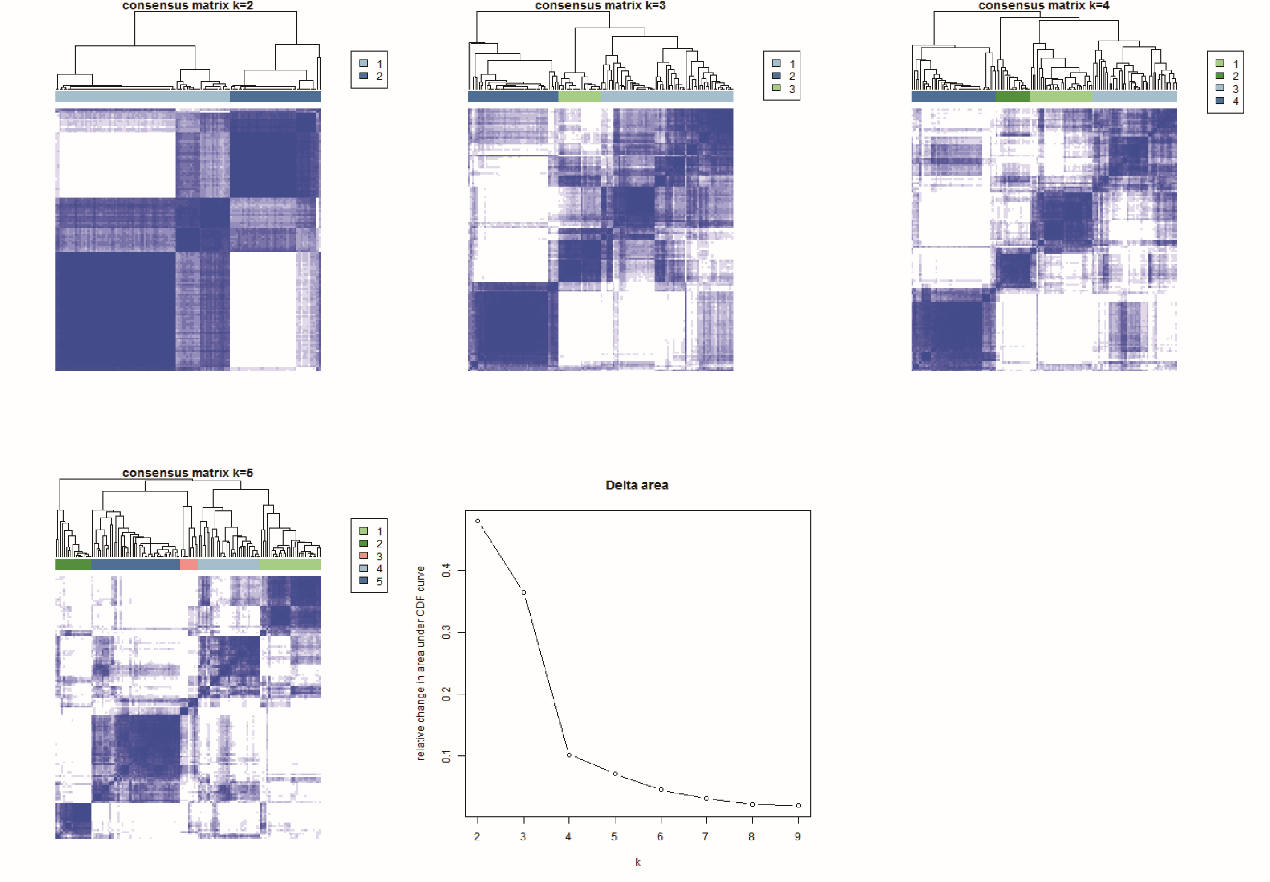


**Fig. S8. Unsupervised clustering of CRGs and consensus matrix heatmaps for k=2-5 in the GSE21501 cohort.**


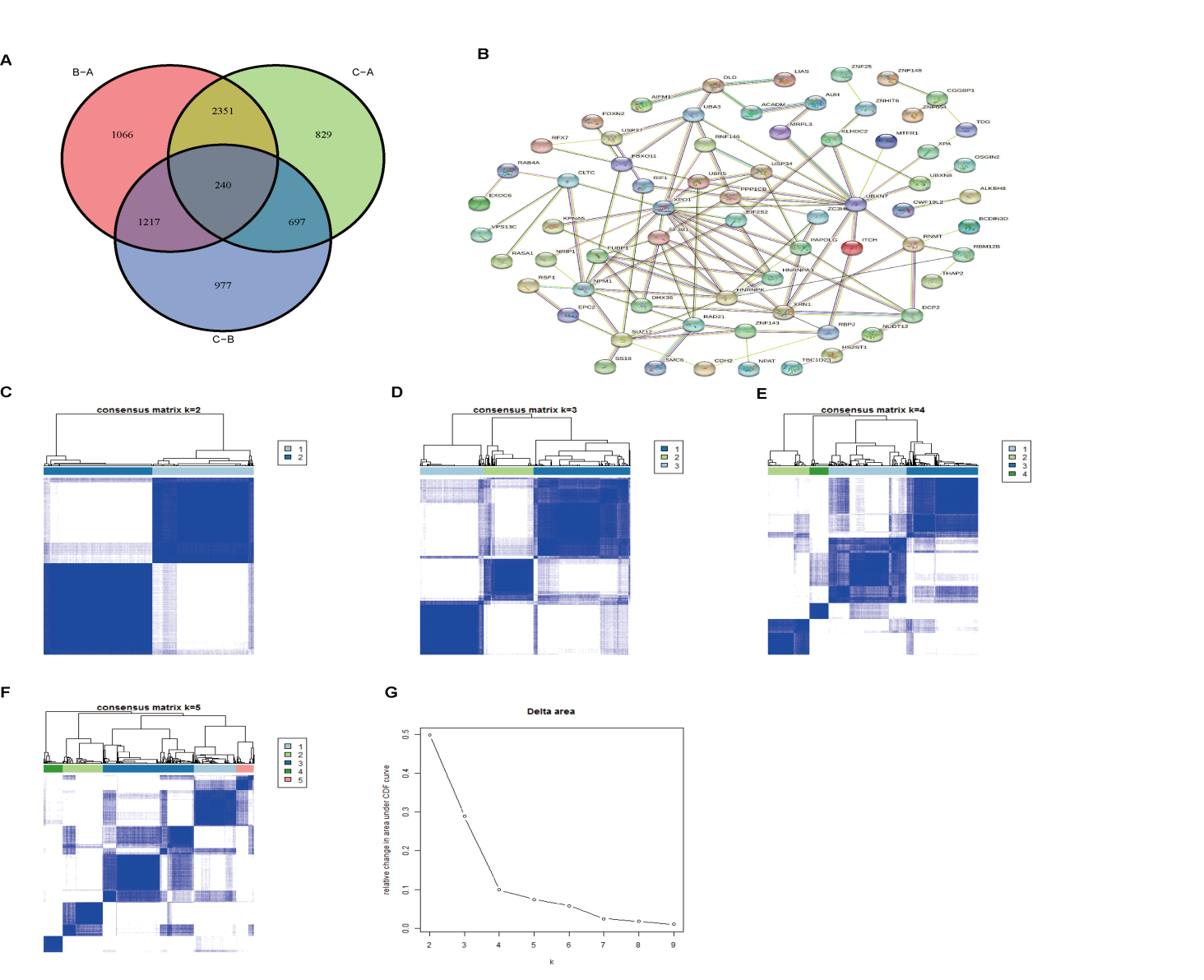


**Fig. S9. Identification of gene clusters based on DEGs.** (**A**) Venn diagram showing the intersection genes among the three pyroptosis clusters. (**B**) The protein‒protein network (PPI) of DEGs based on the STRING database with a combined score >0.40. (**C-G**) Consensus clustering of DEGs in PAAD cohorts and consensus matrices for k=2-5.


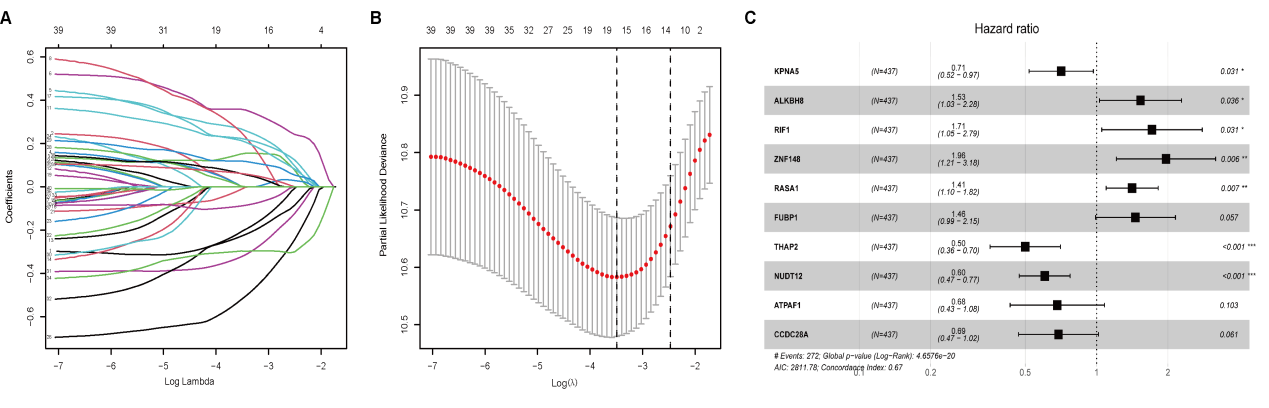


**Fig. S10. Construction of cuproptosis signature.** (**A**) Profiles of LASSO coefficients. (**B**) LASSO penalized Cox regression analysis. The vertical dashed line is at the optimal log (lambda) value. (**C**) Multivariate Cox regression analysis.


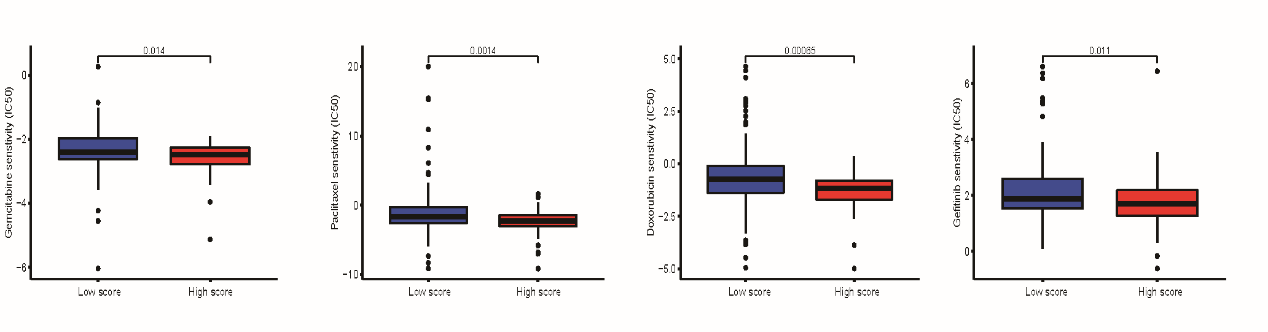


**Fig. S11. Relationships between the cuproptosis score and chemotherapy sensitivity.**

**
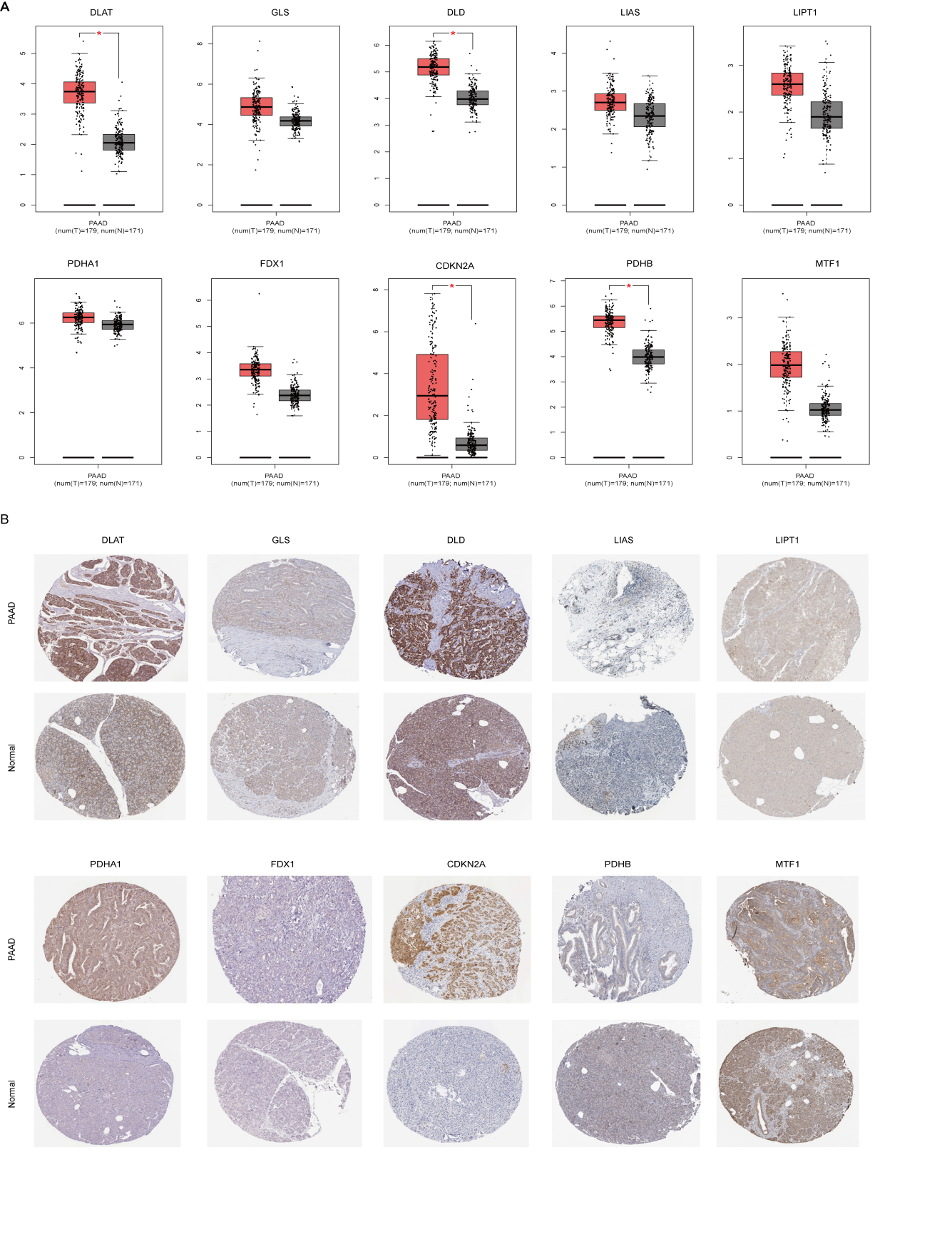
**

**Fig. S12. Comparison of the expression levels of the 10 CRGs in PAAD tissues and normal pancreatic tissues.** (**A**) Comparison of the differential expression of CRGs in the GEPIA database. (**B**) Representative IHC images of CRGs in PAAD and normal pancreatic tissues derived from the HPA database.


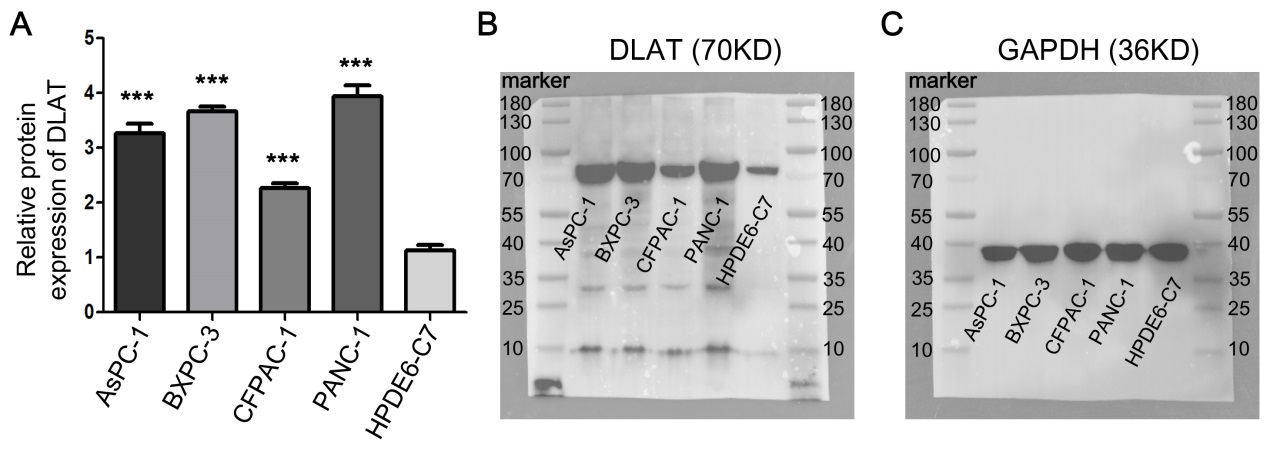


**Fig. S13. Western blot analysis of DLAT protein levels in pancreatic cancer cell lines and normal cells. (A**) The related expression levels of DLAT in AsPC-1, BXPC-3, CFPAC-1, PANC-1 and HPDE6-C7 cell lines, respectively. (**B, C**) Western blot analysis of DLAT and GAPDH in the aforementioned cell lines.
